# Murine cell lines with defined mutations model different histological subtypes of epithelial ovarian cancer

**DOI:** 10.1242/dmm.052177

**Published:** 2025-07-28

**Authors:** Lixin Zhang, Yusi Fang, Ibrahim Uygun, Danyang Li, Mary Strange, Syed K. Zaidi, Wenjia Wang, Julia Knight, Mackenzy Radolec, Esther Elishaev, Joan F. Brozick, Allison Edwards, George Tseng, Sandra Cascio, Ronald Buckanovich, Robert P. Edwards, Anda M. Vlad

**Affiliations:** ^1^ University of Pittsburgh School of Medicine, Department of Obstetrics, Gynecology and Reproductive Sciences, Pittsburgh, PA 15261, USA; ^2^ Magee-Womens Research Institute, Pittsburgh, PA 15213, USA; ^3^ University of Pittsburgh School of Public Health, Department of Biostatistics, Pittsburgh, PA 15261, USA; ^4^ Magee-Womens Hospital of UPMC, Pittsburgh, PA 15213, USA; ^5^ University of Pittsburgh School of Medicine, Department of Pathology, Pittsburgh, PA 15261, USA

**Keywords:** Ovarian surface epithelium, Immortalization, Transformation, *Trp53*, *Kras*, *Pten*, MUC1

## Abstract

Preclinical modeling of epithelial ovarian cancer in immune-competent mice progressing to orthotopic, spontaneous tumors is challenging, requiring multiple genetic modifications in the host. Transplantable models using cell lines are easier to implement than spontaneous animal models, given that they reproduce the key disease characteristics. To create new *in vivo* ovarian tumor models, we generated 28 murine ovarian cancer cell lines with distinct genetic traits, such as deletion of *Trp53*, activation of *Kras^G12D^*, or deletion of *Pten* or *Kras^G12D^/Pten^−/−^* combination. Two distinct *Trp53* null cell lines recapitulate high-grade serous histology when orthotopically injected into immune-competent, syngeneic hosts. Cells with *Pten* deletion trigger high-grade endometrioid tumors, and cells with dual *Kras*^G12D^ activation and *Pten* deletion model carcinosarcoma. The cells express different tumor antigens, secrete varying levels of cytokines and chemokines, and trigger tumors with diverse inflammation profiles and various intratumoral T- and B-lymphocyte infiltration patterns. RNA-sequencing data from 16 cell lines reveal the gene expression profile across distinct models with different histotypes. This versatile collection of murine cell lines supports translationally relevant studies in ovarian cancer.

## Teaser

**Summary:** This study provides genotypic and phenotypic analyses of novel murine cell lines that express different antigens and recapitulate *in vivo* ovarian cancer with different histology and inflammatory profiles.

## INTRODUCTION

Epithelial ovarian cancer (EOC) has distinct histological subtypes with unique genomic characteristics (Cook and Vanderhyden, 2019; Kim et al., 2018; Kurman and Shih Ie, 2008; Lheureux et al., 2019). Endometrioid, clear-cell and mucinous carcinomas tend to be histologically low-grade, slower-growing tumors often associated with variants in genes such as *KRAS*, *BRAF*, *PTEN* and *CTNNB1* (Kurman and Shih Ie, 2008; Malpica and Wong, 2016; Vang et al., 2009). High-grade serous ovarian carcinoma (HGSOC), the most common and most aggressive type of EOC, represents ∼70% of all diagnoses and accounts for 75% of ovarian cancer-related deaths (Labidi-Galy et al., 2017; Lisio et al., 2019). Cases of HGSOC are diagnosed at a late stage, and, despite aggressive surgery and chemotherapy, most patients will later relapse and die from treatment-resistant disease. There is a crucial need for the development of new therapeutic venues for EOC, and progress partly depends on the development of preclinical models that adequately reproduce key features of the human disease, including genetic frame, tissue heterogeneity and syngeneic immune microenvironment.

The source of HGSOC is predominantly the secretory fallopian tube epithelium (Meserve et al., 2017; Zhang et al., 2019). Perets et al. (2013) showed that inactivation of *Brca1/2*, *Trp53* and *Pten* in Pax8-expressing murine tubal secretory cells induced serous tubal intraepithelial carcinoma (STIC) lesions. Over time, the STIC lesions advanced to aggressive tumors that spread to the ovary and peritoneum (Perets et al., 2013). Similarly, genetic inactivation of various combinations of *Brca1*, *Trp53*, *Rb1* and *Nf1* in Ovgp1-expressing tubal epithelial cells leads to high-grade serous carcinomas that arise in the mouse oviduct (Zhai et al., 2017).

Although the tubal fimbria is considered to be the site of origin for HGSOC in women (Labidi-Galy et al., 2017; Lee et al., 2007; Meserve et al., 2017), evidence from mouse models shows that loss of *Lkb1* (also known as *Stk11*) and *Pten* in the ovarian surface epithelium (OSE) can also trigger HGSOC with 100% penetrance (Tanwar et al., 2014). Additionally, individual expression of *Hoxa9*, *Hoxa10* and *Hoxa11* in the OSE leads to transformation and triggers orthotopic ovarian carcinoma that resembles serous, endometrioid and mucinous EOC, respectively (Cheng et al., 2005; Connolly et al., 2003; Dinulescu et al., 2005; Fan et al., 2009; Flesken-Nikitin et al., 2003; Orsulic et al., 2002; Wu et al., 2007).

The advent of genetically modified mice progressing to *de novo*, orthotopic EOC has fostered groundbreaking research on ovarian tumor initiation, growth and metastasis (Le Bras, 2024). These mouse models also constitute versatile tools to study interactions between cancer cells and non-malignant stromal cells – such as endothelial cells, fibroblasts, adipocytes and immune cells. However, owing to several disadvantages, such as complex breeding strategies, long disease latency and cost, the adoption of these models has been somewhat constrained. To circumvent some of these limitations, *in vivo* studies often employ transplantable tumor models based on syngeneic ovarian cancer cell lines, with the OSE-derived ID8 model in C57BL/6 mice being the most widely used to date (Roby et al., 2000). Because the original ID8 cells lack relevant mutations, ID8-derived clones with mutations in *Trp53*, *Brca1*, *Brca2*, *Pten* and *Nf1* have been generated and serve as improved *in vivo* models of HGSOC in C57BL/6 mice (Cook et al., 2023; Galpin et al., 2024; Walton et al., 2016, 2017).

To enhance the range of *in vitro* and *in vivo* models for EOC, we have developed a significant collection of new murine cell lines that replicate key characteristics of the disease. Through genetic engineering, these cell lines exhibit various molecular traits, making them versatile tools for studying genes critical to ovarian cancer biology, such as *Trp53*, *Kras* and *Pten*. Some of these models also express human mucin 1 (*MUC1*) as a transgene. MUC1 oncoprotein is a tumor-associated antigen, overexpressed by most epithelial ovarian tumors (Chen et al., 2024; Kufe, 2013). Somatic variants in *MUC1* are rare in ovarian cancer, occurring in less than 1% of cases (Cancer Genome Atlas Research, 2011). However, *MUC1* overexpression has been observed to co-occur with driver gene variants such as *TP53*, *KRAS* and *PTEN* in ovarian and other cancer types (Kharbanda et al., 2014; Kloudova et al., 2016; Kufe, 2013; L'Esperance et al., 2006; Rajabi et al., 2018). MUC1 glycoprotein is also a widely validated immune therapy target that can be tested in fully syngeneic *MUC1* transgenic mice, which mirror the anatomical expression seen in humans (Budiu et al., 2013; Deng et al., 2013; Tirodkar et al., 2014). We detail here the transcriptomic profiles of several new EOC mouse models and analyze their *in vivo* tumor growth kinetics, cytokine and chemokine secretion, and immune phenotypes to guide their adoption for translational investigations in EOC.

## RESULTS

### Generation of new ovarian cancer cell lines with defined genetic traits, via *in vitro* spontaneous immortalization of primary OSE

**Fig. 1.**
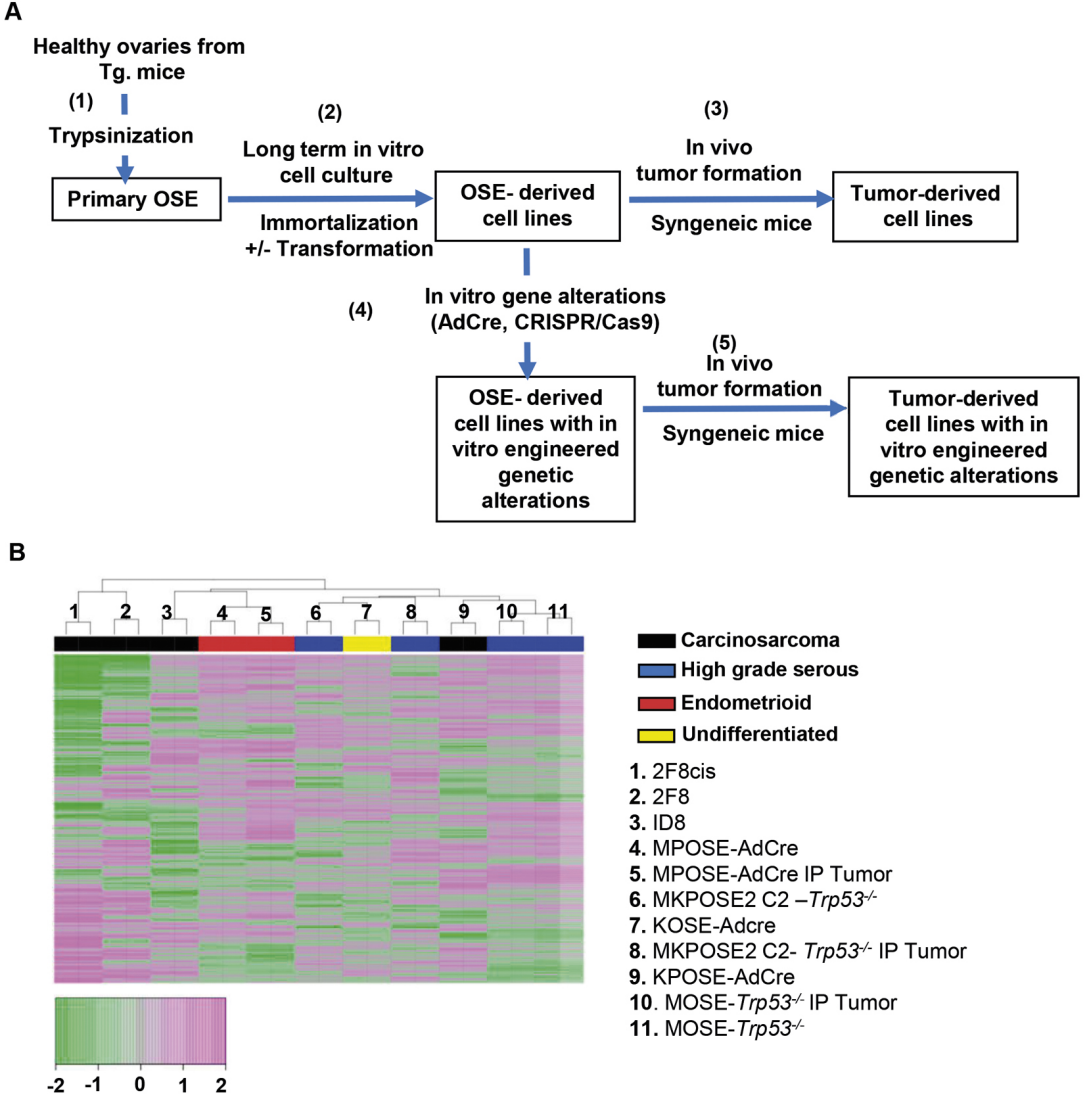
**Murine ovarian cancer cell line generation protocols.** (A) Generation of cell lines using healthy OSE cells isolated from mice with specific genetic traits. Following gentle trypsinization (1), primary OSE cells were cultured *in vitro* for prolonged periods of time (2), until immortalization was achieved, as detailed in the Materials and Methods. Cells that also underwent transformation were used as transplantable tumor models in syngeneic mice (3). Tumor tissue isolated at necropsy was used to generate tumor-derived cell lines. Cells were further used for *in vitro* genetic manipulations (4), such as via activation of silent Cre/loxP mutations or gene inactivation via CRISPR/Cas9. These newly derived ovarian cancer cell lines, with modified genetic traits, can also be used for *in vivo* tumor formation (5) and subsequent isolation of tumor-derived, ‘daughter’ cell lines. (B) Heatmap using normalized gene expression (R package DESeq2) across 11 transformed cell lines, run as duplicates. Color coding represents histology, as shown. AdCre, Cre-encoding adenovirus; OSE, ovarian surface epithelium; Tg, transgenic.

The overall strategy we implemented to generate new murine ovarian cancer cell lines is presented in Fig. 1, and the genetic traits of each cell line are listed in Table 1. Primary OSE obtained via gentle trypsinization of healthy ovaries from mice with various genetic backgrounds were propagated long term *in vitro*, until stable cell lines were obtained (Fig. 1A). This previously described approach renders a homogenous population of primary OSE cells, with few/no contaminant cells present (Roby et al., 2000). Following prolonged *in vitro* passaging, immortalization was obtained in a total of eight OSE cell populations (KOSE, POSE, KPOSE, MOSE, MKOSE, MPOSE, MKPOSE2, MKPOSE2-C2; Table 1). The cell lines were further genetically modified to carry, alone or in combination, homozygous deletion of *Trp53* (MOSE-*Trp53^−/−^*, MKPOSE2-C2-*Trp53^−/−^*, KPOSE-*Trp53^−/−^*), conditional inactivating deletion of *Pten*, and/or conditional activating mutation in oncogenic *Kras^G12D^* (KOSE-AdCre, POSE-AdCre, KPOSE-AdCre, MKOSE-AdCre, MPOSE-AdCre, MKPOSE2-AdCre) (Table 1). As indicated by the letter ‘M’ in each individual name, some of the cell lines have been derived from mice carrying human *MUC1* as a transgene (Budiu et al., 2013). The distinct genetic traits of all our newly obtained murine ovarian cancer cell lines, derived from single, double or triple transgenic mice, are detailed in Table 1. The average time to immortalization for all cell lines was 243 days (median, 260 days; range 162-350 days).

**Table 1.** Characteristics of ovarian surface epithelium and tumor-derived murine ovarian cancer cell lines

#	Cell line	Genetic traits	Pathway activation*	Colony formation assay^‡^	*In vivo* tumor^§^	Concentrations (pg/ml)^¶^						
						Eotaxin (CCL11)	IP-10 (CXCL10)	MIP-2 (CXCL2)	RANTES (CCL5)	TNF-α	VEGF	MCP-1 (CCL2)
1	KOSE	LSL-*Kras^G12D/+^* (silent)		N	Not transformed	470.64	30.47	29.32	6.09	0.78	10.78	2096.76
2	KOSE-AdCre	*Kras^G12D/+^*	Increased pMEK		High-grade undifferentiated	480.88	40.67	9.69	12.43	0.72	4.34	1856.20
3	K-Ad-ASC**	*Kras^G12D/+^*	−		N/A	253.01	24.51	19.58	2.55	0.35	2.20	1472.85
4	K-Ad-IPT**	*Kras^G12D/+^*	−		N/A	182.63	14.77	32.28	3.40	0.53	3.48	1159.42
5	POSE	*Pten^loxP/loxP^ *(silent)	−	N	Not transformed	661.08	68.27	4.23	768.02	0.51	0.74	2060.48
6	POSE-AdCre	*Pten^−/−^*	Increased pAKT		N/A	210.58	46.05	8.10	233.22	0.43	4.30	1173.96
7	KPOSE	LSL*-Kras^G12D^* (silent) *Pten^loxP/loxP^* (silent)		N	Not transformed	474.61	61.37	1.39	3.25	0.85	64.68	1603.74
8	KPOSE-AdCre	*Kras^G12D/+^ Pten^−/−^*	Increased pMEK, pAKT		Carcinosarcoma (SC)	700.29	61.32	1.51	4.91	0.94	110.12	1938.08
9	KP-Ad-SC^‡‡^	* Kras^G12D/+^ Pten^−/−^*	Increased pMEK, pAKT		N/A	321.73	136.38	260.42	12.36	8.87	857.05	1408.92
10	MKOSE	*MUC1^+/−^* LSL*-Kras^G12D^* (silent)	−	N	Not transformed	493.20	34.40	20.96	15.49	0.80	5.77	1635.25
11	MKOSE-AdCre	*MUC1^+/−^ Kras^G12D/+^*	Increased pMEK		Low-grade serous	485.46	69.63	34.55	2.71	0.67	2.46	1632.98
12	MPOSE	*MUC1^+/−^ Pten^loxP/loxP^* (silent)	−	Y	High-grade endometrioid	1317.97	81.33	72.75	22.62	0.76	4.92	2355.35
13	MPOSE-AdCre	*MUC1^+/−^ Pten^−/−^*	Increased pAKT		High-grade endometrioid	1273.47	221.03	87.78	85.59	1.05	12.32	2125.09
14	M3230-IP^§§^	*MUC1^+/−^ Pten^loxP/loxP^* (silent)	−		High-grade endometrioid	1105.00	260.98	141.10	131.38	23.53	6.24	1996.93
15	M3230-Asc^§§^	*MUC1^+/−^ Pten^loxP/loxP^* (silent)	−		High-grade endometrioid	1118.00	351.16	180.21	143.53	22.67	7.27	2012.23
16	MKPOSE2	*MUC1^+/−^* LSL*-Kras^G12D/+^* (silent) *Pten^loxP/loxP^* (silent)	−	N	Not transformed	148.28	33.41	36.93	36.83	0.79	4.36	950.97
17	MKPOSE2-AdCre	*MUC1^+/−^ Kras^G12D/+^* *Pten^−/−^*	Increased pMEK, pAKT		High-grade undifferentiated	8.80	15.38	17.56	30.29	3.06	912.43	95.90
18	KPOSE-*Trp53*^−/−^	LSL-*Kras^G12D/+^* (silent) *Pten^loxP/loxP^* (silent) *Trp53^−/−^*	p53 KO		Not transformed	152.77	66.88	2.19	12.02	0.20	134.49	880.98
19	MOSE	*MUC1^+/−^*	−	N	Not transformed	1040.38	95.58	25.96	15.33	2.03	362.52	1811.89
20	MOSE-*Trp53*^−/−^	*MUC1^+/−^*	P53 KO		N/A	1139.85	79.31	64.77	55.31	3.44	325.31	2127.44
21	MOSE-*Trp53*^−/−^- C1	*MUC1^+/−^ Trp53^−/−^*	P53 KO		High-grade serous	1047.10	125.98	128.78	22.66	1.76	999.81	1789.31
22	MOSE-*Trp53*^−/−^-IP	*MUC1^+/−^ Trp53^−/−^*	P53 KO		High-grade serous	1177.47	127.41	66.86	41.36	1.42	306.48	1874.68
23	MOSE-*Trp53*^−/−^-Lung	*MUC1^+/−^ Trp53^−/−^*	P53 KO		High-grade serous	1264.93	276.25	68.15	60.76	1.28	397.53	1835.82
24	MKPOSE2-C2	*MUC1^+/−^* LSL*-Kras^G12D^* (silent) *Pten^loxP/loxP^* (silent)	−	N	Not transformed	141.37	16.01	37.53	7.97	1.24	0.54	647.61
25	MKPOSE2-C2- *Trp53*^−/−^	*MUC1^+/−^ Trp53^−/−^* LSL*-Kras^G12D^* (silent) *Pten^loxP/loxP^* (silent)	P53 KO		High-grade serous	70.59	9.75	49.18	9.55	1.40	0.15	474.99
26	MKPOSE2-C2- *Trp53*^−/−^-C7	*MUC1^+/−^ Trp53^−/−^* LSL*-Kras^G12D^* (silent) *Pten^loxP/loxP^* (silent)	P53 KO		High-grade serous	557.13	33.32	50.06	7.07	1.94	1.24	1195.32
27	MKPOSE2-C2- *Trp53*^−/−^-IP	*MUC1^+/−^ Trp53^−/−^* LSL*-Kras^G12D^* (silent) *Pten^loxP/loxP^* (silent)	P53 KO		High-grade serous	46.00	20.50	20.13	1.82	0.53	7.80	309.72
28	MKPOSE2-C2- *Trp53*^−/−^-Lung	*MUC1^+/−^ Trp53^−/−^* LSL*-Kras^G12D^* (silent) *Pten^loxP/loxP^* (silent)	P53 KO		High-grade serous	630.85	214.00	129.20	29.55	0.73	6.01	1228.27
29	2F8	*MUC1^+/−^ Kras^G12D/+^* *Pten^−/−^*	Increased pMEK, pAKT		Carcinosarcoma (IP and SC)	914.69	163.80	1.12	49.46	0.76	3.83	1628.91
30	2F8cis	*MUC1^+/−^ Kras^G12D/+^* *Pten^−/−^*	Increased pMEK, pAKT		Carcinosarcoma (IP and SC)	1070.35	219.32	2.37	571.16	0.94	34.07	1610.71
31	ID8^¶¶^	No engineered mutations	−		Carcinosarcoma	108.36	106.98	0.84	123.93	0.41	297.48	1492.27
32	ID8-VEGF^¶¶^	No engineered mutations	−		Carcinosarcoma	272.38	79.72	0.86	127.28	0.62	1516.55	1574.91

*Pathway activation triggered through Cre-loxP recombination, post exposure *in vitro* or *in vivo* to AdCre.

^‡^Colony formation assay results: N, negative; Y, positive. For cell lines without displayed results, the assay was not conducted.

^§^Histological appearance of tumors triggered by IP injection of the respective cell lines. Cell lines #8, #29 and #30 have also been injected SC.

^¶^Concentrations (pg/ml) of eotaxin, IP-10, MIP-2, RANTES, TNFα and VEGF were measured by MSD. MCP-1 was measured by ELISA.

**K-Ad-ASC (#3) and K-Ad-IPT (#4) cell lines have been derived from tumors ascites and IP tumor nodules, respectively, isolated at necropsy post IP injection of KOSE-AdCre cells (#2).

^‡‡^KP-Ad-SC cell line (#9) has been derived from a SC tumor nodule isolated at necropsy post SC injection of KPOSE-AdCre cells (#8).

^§§^M3230-IP (#14) and M3230-Asc (#15) cell lines have been derived from one IP tumor nodule and ascites cells, respectively, post IP injection of MPOSE-AdCre (#13) cell line in mouse M3230.

^¶¶^ID8 and ID8-VEGF have been described elsewhere (Roby et al., 2000; Zhang et al., 2002) and were included here as reference.

AdCre, Cre-encoding adenovirus; ELISA, enzyme-linked immunosorbent assay; IP, intraperitoneal; KO, knockout; MSD, Meso Scale Discovery; N/A, not available (*in vivo* tumor growth was not tested); SC, subcutaneous.

We also attempted to generate cell lines from mouse oviduct epithelia, using a similar *in vitro* passaging protocol. Surprisingly, immortalization of these primary cells proved technically more challenging, requiring nearly 20 months of continuous *in vitro* passaging before the oviduct cells became immortalized (MPDuct cell line, Fig. S1). As a result, we continued to focus on OSE cells. All lines have been steadily maintained in the laboratory under standard cell growth conditions and monitored for several *in vitro* passages. Cellular transformation was assessed using colony formation in soft agar. *In vivo* tumor growth of colony-forming transformed lines was measured mainly upon injection into syngeneic hosts. As a surrogate for orthotopic growth, the intraperitoneal (IP) route has been the preferred approach in all our experiments. To assess more rapidly the tumorigenic capacity of the new cell lines, we have at times used both IP and subcutaneous (SC) injections, which also allowed us to evaluate tumor growth under different microenvironmental conditions. As shown in Table 1, this approach has (unsurprisingly) revealed that some cell lines (2F8 and 2F8cis) can form tumors in both environments, while others show a preference for a specific milieu. Tumor tissue or ascites cells isolated at necropsy were further used for the generation of cell lines (Fig. 1A). As expected, compared to the primary OSE-derived ‘parental’ cells, the *in vivo*-passaged, tumor tissue-derived ‘daughter’ cells carry new phenotypic characteristics, imprinted by *in vivo* growth, such as shorter time to progression, as further detailed below.

Altogether, we generated a total of 28 cell lines, which carry a variety of genetic modifications and histologically recreate several different EOC types, including the high-grade serous, high-grade endometrioid and carcinosarcoma histotypes (Table 1). To characterize these new models, we performed whole-transcriptome RNA sequencing (RNAseq) on most representative (*n*=16) cell lines (Fig. S2A). As reference, we included the widely used ID8 cells (Roby et al., 2000). We also included our previously reported 2F8 and 2F8cis cell lines (Grabosch et al., 2019; Mony et al., 2015; Zhang et al., 2016a), used as platinum-sensitive and platinum-resistant carcinosarcoma models, respectively. A gene expression heatmap with hierarchical clustering of the 11 cell lines that trigger tumors *in vivo* shows that the cell lines largely cluster according to their respective histotype (Fig. 1B).

We have also used the RNAseq data for analyses of copy number variations (CNVs) (Serin Harmanci et al., 2020). Using as reference the mRNA profiles of normal mouse ovaries from five healthy mice, we identified CNVs in all cell populations (Fig. S2B). In line with the human disease, which frequently exhibits gene number aberrations, the most common alteration across the murine cell lines was gene amplification, identified in chromosomes 7 to 19, while deletions occurred mainly in chromosomes 1 to 3 (Fig. S2B). As expected, *in vitro* activation of oncogenic pathways leads to additional genetic aberrations. Similarly, and perhaps most strikingly, exposure to cisplatin revealed additional aberrations, with increased alterations observed in platinum-resistant 2F8cis cells compared to the parental, platinum-sensitive 2F8 cells (Fig. S2C).

### Deletion of *Trp53* triggers OSE transformation and leads to ovarian tumors with high-grade serous histology

**Fig. 2.**
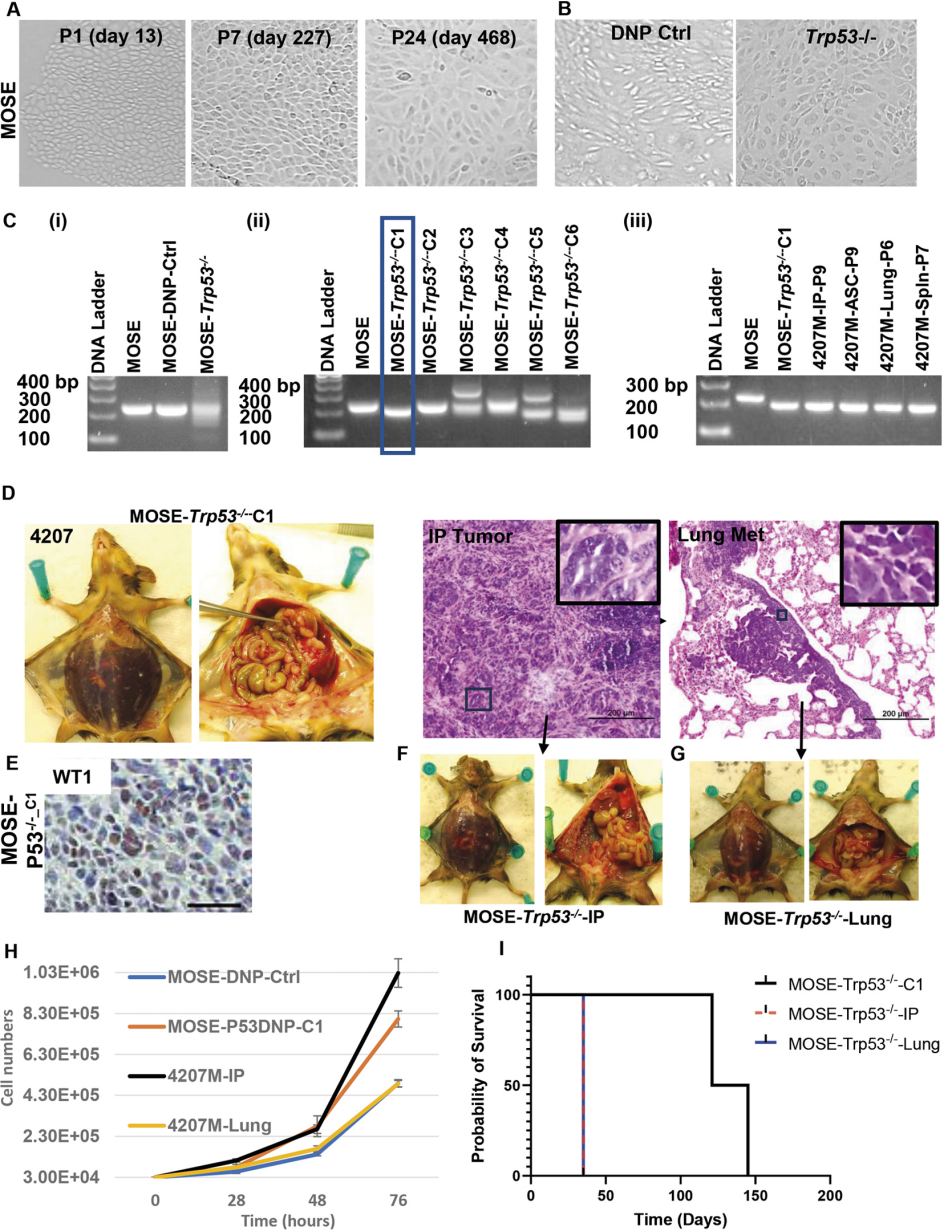
***Trp53* deletion supports cell transformation and leads to ovarian tumors with high-grade serous histology.** (A) Progression to spontaneous immortalization of MOSE cells during prolonged *in vitro* passaging. A stable cell line was obtained after 277 days (16 passages) in culture. Changes in cell morphology at early (day 13), intermediate (day 227) and late (day 468) time points are shown from left to right, respectively. P, postnatal day. (B) Once immortalized, the MOSE cells were modified with CRISPR/Cas9 to functionally delete *Trp53* (right). Control, double nickase plasmid (DNP)-treated cells are shown on the left. (C) Confirmation of *Trp53* deletion via PCR. (i) Parental (non-treated) and control DNP-treated MOSE cells carry wild-type *Trp53*. Exposure to DNP targeting exon 5 of *Trp53* leads to a mixture of cells with varying degrees of target-site cutting and self-repair of *Trp53*, shown as a band smear. (ii) Bulk cells exposed to *Trp53*-specific DNP were subjected to limiting dilution, and single clones were selected. Clone 1 (rectangle) shows a single, lower band, suggestive of homozygous *Trp53* deletion, and was selected for further *in vivo* studies. (iii) PCR confirming that following *in vivo* injection of clone 1 into syngeneic mouse (4207) tumor cells isolated from three different anatomic locations [intraperitoneal (IP) tumor nodule, ascites, lung or spleen metastasis] show the same band, suggesting *in vivo* clonal expansion. (D) IP injection of 5 million MOSE-*Trp53*^−/−^*-*C1 cells into mice leads to widespread peritoneal tumors accompanied by hemorrhagic ascites and hematogenous metastases to the lung. One representative mouse (4207) of three injected mice is shown. Hematoxylin and Eosin (HE) images of one IP tumor nodule (left) and lung metastasis (lung met; right) are shown in the right panel. The tumors show high-grade serous histology, as further described in Fig. 6. The insets in the upper-right corner are from the fields indicated with black outline boxes. (E) Immunohistochemistry staining for WT1 (brown) in MOSE-*Trp53*^−/−^ IP tumor nodule. Scale bar: 50 µM. (F) The IP tumor from mouse 4207 was isolated and tumor passaged *in vitro* until cells were stably maintained. The resultant cell line (MOSE-*Trp53*^−/−^-IP) was injected IP into three syngeneic mice. All injected mice developed widespread peritoneal disease and ascites. Progression to end-stage disease occurred in 35 days. One mouse, representative of the group, is shown before and after peritoneal incision. (G) The lung tumor nodule isolated from mouse 4207 was isolated and tumor cells passaged *in vitro* until cells were stably maintained. The resultant cell line (MOSE-*Trp53*^−/−^-Lung) was injected IP into three syngeneic mice. All injected mice developed widespread peritoneal disease and ascites. Humane endpoints were reached after an average of 35 days. One mouse, representative of the group, is shown, before and after peritoneal incision. (H) *In vitro* growth curves of control and *Trp53* null derivatives of MPOSE cell line. (I) Survival curves of MOSE-*Trp53*^−/−^*-*C1, MOSE-*Trp53*^−/−^-IP and MOSE-*Trp53*^−/−^-Lung cells in syngeneic mice (three mice per group).

Using the approach described in Fig. 1A, the MOSE cell line was obtained after 277 days in culture. During early passages, the cells multiplied slowly and displayed the typical cobblestone morphology (Fig. 2A). In time, cells progressively acquired faster growth rates and underwent morphology changes, with cells becoming more spindle shaped, often indicating epithelial-to-mesenchymal transition (EMT) (Fig. 2A) (Kalluri, 2009; Zhang et al., 2016b). Despite being immortalized, the MOSE cells do not form colonies when plated on soft agar and do not grow tumor *in vivo* 5 months after IP tumor challenge of syngeneic mice, suggesting that they are not transformed.

However, transformation was achieved following functional deletion of *Trp53*, using CRISPR/Cas9-mediated removal of exon 5 (Fig. 2B). PCR analyses of viable cells post-selection show that *Trp53* deletion leads to a mixture of cells with varying degrees of *Trp53* loss (Fig. 2Ci). Following separation into single clones via limiting dilution, we isolated and further characterized several monoclonal populations. Of these, clone 1 (MOSE-p53^−/−^-C1) showed homozygous *Trp53* deletion (Fig. 2Cii) and was selected for further *in vivo* studies. DNA sequencing of MOSE-p53^−/−^-C1 confirmed the targeted deletion and predicted a protein truncated to 159 amino acids (compared to 390 amino acids in the wild-type sequence) (Fig. S3A).

IP injection of 5×10^6^ MOSE-*Trp53^−/−^-*C1 cells into three syngeneic hosts led to widespread peritoneal tumors, hemorrhagic ascites and hematogenous metastases to the lung (Fig. 2D). After 90 days, there were no visible signs of tumor growth, confirmed by sacrificing one of the mice. However, with extended monitoring, progression to end-stage disease occurred in the remaining two mice at an average of 133 days (121 and 145 days, respectively), both of which developed lung metastases. Importantly, the tumors were WT1 positive and exhibited histomorphology consistent with high-grade serous cancer, most frequently seen in patients (Fig. 2E) (Magrill et al., 2019). Tumor cells isolated from different anatomic locations (IP tumor nodule, ascites, lung and spleen metastases) show the same gene rearrangement at the *Trp53* locus, confirming *in vivo* clonal expansion (Fig. 2Ciii). Tissues from one IP tumor nodule and one metastatic lesion to the lung were further used for *in vitro* propagation, until two stable cell lines were obtained (MOSE-*Trp53^−/−^*-IP and MOSE-*Trp53^−/−^*-Lung, respectively; Table 1). When injected IP, these cells trigger a tumor phenotype that is like the one observed with ‘parental’ MOSE-*Trp53^−/−^*-C1 (Table 1), characterized by large tumor burden with high-grade serous histology and hemorrhagic ascites (Fig. 2F,G). The *in vitro* proliferation rates were similar for all cell lines, with doubling times for control double nickase plasmid (DNP)-treated MOSE-DNP-Ctrl, MOSE-*Trp53^−/−^-*C1, MOSE-*Trp53^−/−^*-IP and MOSE-*Trp53^−/−^*-Lung cells of 16.68 h, 15.45 h, 14.59 h and 17.63 h, respectively, Fig. 2H). However, progression to end-stage disease occurred much faster after injection of three mice with cell lines obtained after *in vivo* passaging: 35 days for *in vivo*-passaged, tumor-derived MOSE*-Trp53^−/−^*-IP and MOSE-*Trp53^−/−^*-Lung cell line, compared to 133 days for the ‘parental’ *in vitro*-generated MOSE-*Trp53^−/−^-*C1 cells (Fig. 2I).

**Fig. 3.**
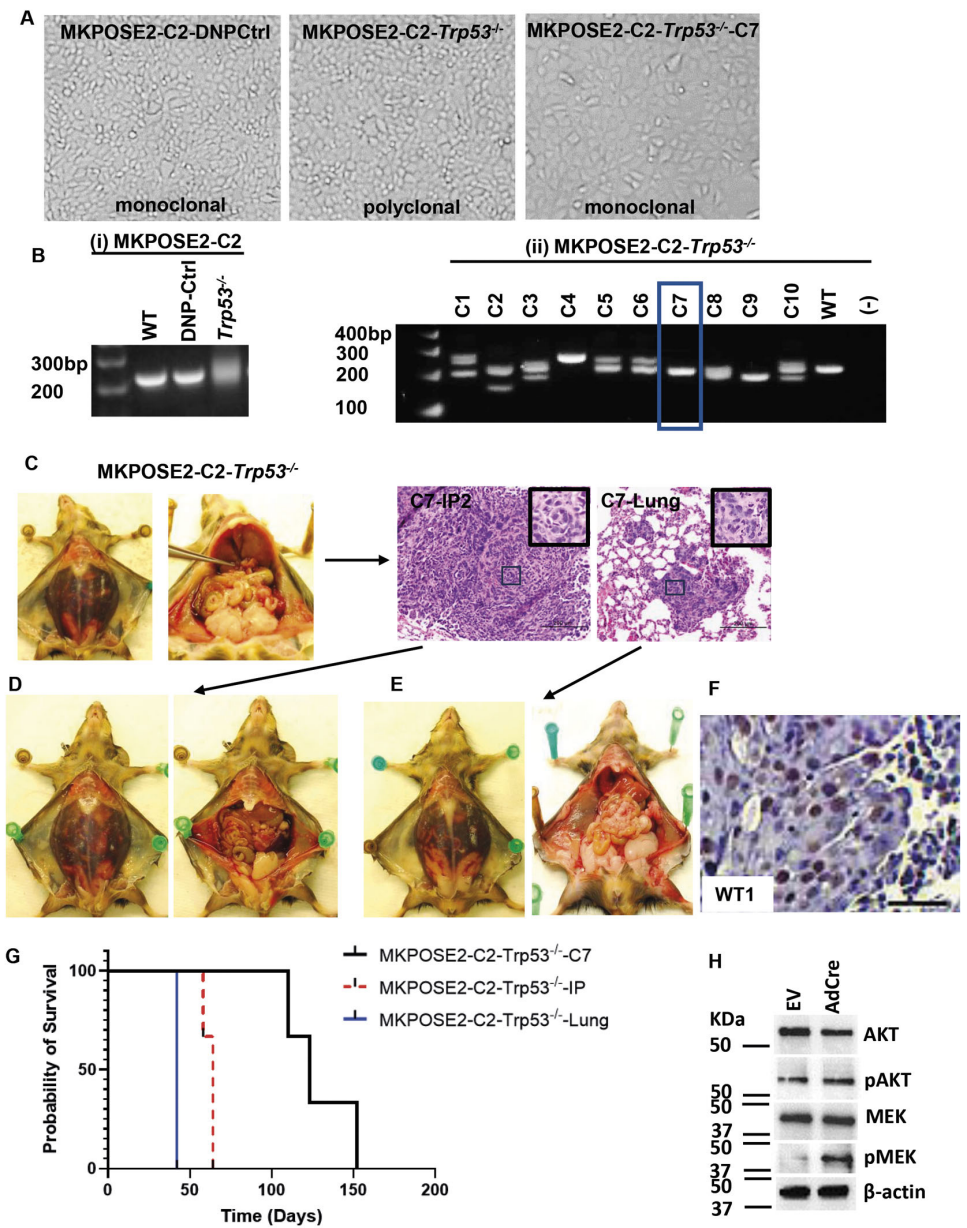
**Generation of a triple transgenic cell line with Trp53 deletion and silent conditional (Cre-loxP) loxP-Stop-loxP-Kras^G12D/+^ and Pten^loxP/loxP^ mutations.** (A) Monoclonal, immortalized MKPOSE2-C2 cells were modified with CRISPR/Cas9 to functionally delete *Trp53* (middle). Control, DNP-treated cells are shown on the left. Limiting dilution was used to select single clones. One of these clones (clone 7) is shown on the right. (B) Confirmation of *Trp53* deletion via PCR. (i) Parental (non-treated) and control DNP-treated MKPOSE2-C2 cells carry wild-type *Trp53*. Exposure to DNP targeting *Trp53* leads to a mixture of cells with varying degrees of *Trp53* deletion, which appears as a smear due to self-repair of the cutting sites. (ii) Bulk cells exposed to *Trp53* DNP were subjected to limiting dilution, and single clones were selected. Clone 7 (rectangle) shows a single, lower band, suggestive of homozygous *Trp53* deletion, and was selected for further *in vivo* studies. (C) IP injection of 5 million MKPOSE2-C2-*Trp53*^−/−^*-*C7 cells into *n*=3 mice triggers tumors with widespread peritoneal disease accompanied by hemorrhagic ascites and hematogenous metastases to the lung. Progression to end-stage disease occurred after a median of 128 days. One mouse, representative of the group, is shown. HE images of one IP tumor nodule (left) and lung met (right) are shown in the right panel. The insets in the upper-right corner are from the fields indicated with black outline boxes. The tumors show high-grade serous histology, as further shown in Fig. 6A. (D,E) Tumor burden after injection of cell lines generated from the IP tumor nodule (D) and lung met (E) are shown. (F) WT1 staining by immunohistochemistry (IHC) of MKPOSE2-C2-*Trp53*^−/−^ IP tumor nodule. Scale bar: 50 µm. (G) Survival curves of MKPOSE-C2-*Trp53*^−/−^-C7, MKPOSE-C2-*Trp53*^−/−^-C7-IP and MKPOSE-C2-*Trp53*^−/−^-C7-Lung cells in three, three and four syngeneic mice, respectively. (H) Exposure of MKPOSE-C2-*Trp53*^−/−^-C7 cells to AdCre *in vitro* leads to activation of oncogenic Kras and deletion of Pten, resulting in the upregulation of pMEK and pAKT, respectively.

Using the same approach, we deleted *Trp53* in a second cell line [MKPOSE2-C2, previously described by us (Zhang et al., 2016a)] (Fig. 3A). Limiting dilution was used to select single clones (Fig. 3B). Clone 7 (full name MKPOSE2-C2-*Trp53^−/−^-*C7) was confirmed by DNA sequencing to have a homozygous deletion at the *Trp53* locus and a predicted protein size truncated to 172 amino acids (Fig. S3B). This clonal population is fully transformed, and IP injection of 5 million MKPOSE2-C2-*Trp53^−/−^*-C7 cells into three syngeneic mice led to WT1-positive high-grade serous tumors with aggressive peritoneal spread, accompanied by hemorrhagic ascites and hematogenous metastases to the lungs in all three mice (Fig. 3C,F). Progression to end-stage disease occurred after an average of 128 days in syngeneic mice (110, 123 and 152 days, respectively). Primary tissue from peritoneal and lung tumor metastasis nodules were used to develop two stable cell lines (MKPOSE2-C2-*Trp53^−/−^*-IP and MKPOSE2-C2-*Trp53^−/−^*-Lung, respectively; Table 1). When used for tumor challenge, progression to end-stage disease was faster, taking an average of 62 days for MKPOSE2-C2-*Trp53^−/−^*-IP cells injected intraperitoneally into three syngeneic mice (58, 64 and 64 days, respectively) (Fig. 3D,G) and 42 days in all four syngeneic mice for MKPOSE2-C2-*Trp53^−/−^*-Lung (Fig. 3E,G).

Despite both carrying homozygous *Trp53* deletion, and similarly reproducing HGSOC histology *in vivo*, these two models have unique genetic characteristics. Unlike the MOSE-*Trp53^−/−^* cells (Fig. 2) derived from single MUC1 transgenic mice, the MKPOSE2-C2- *Trp53^−/−^* cells (Fig. 3) were derived from the OSE of triple transgenic mice that also carry silent (*loxP*) mutations at the oncogenic *Kras^G12D/+^* and *Pten* (exon 5) loci. Exposure of these cells to Cre-encoding adenovirus (AdCre) *in vitro* leads to simultaneous activation of oncogenic *Kras^G12D^* and deletion of tumor suppressor *Pten*, resulting in increased pathway activation, as demonstrated by the phosphorylated (p)MEK (also known as MAP2K)/MEK and pAKT/AKT ratios (Fig. 3H).

**Fig. 4.**
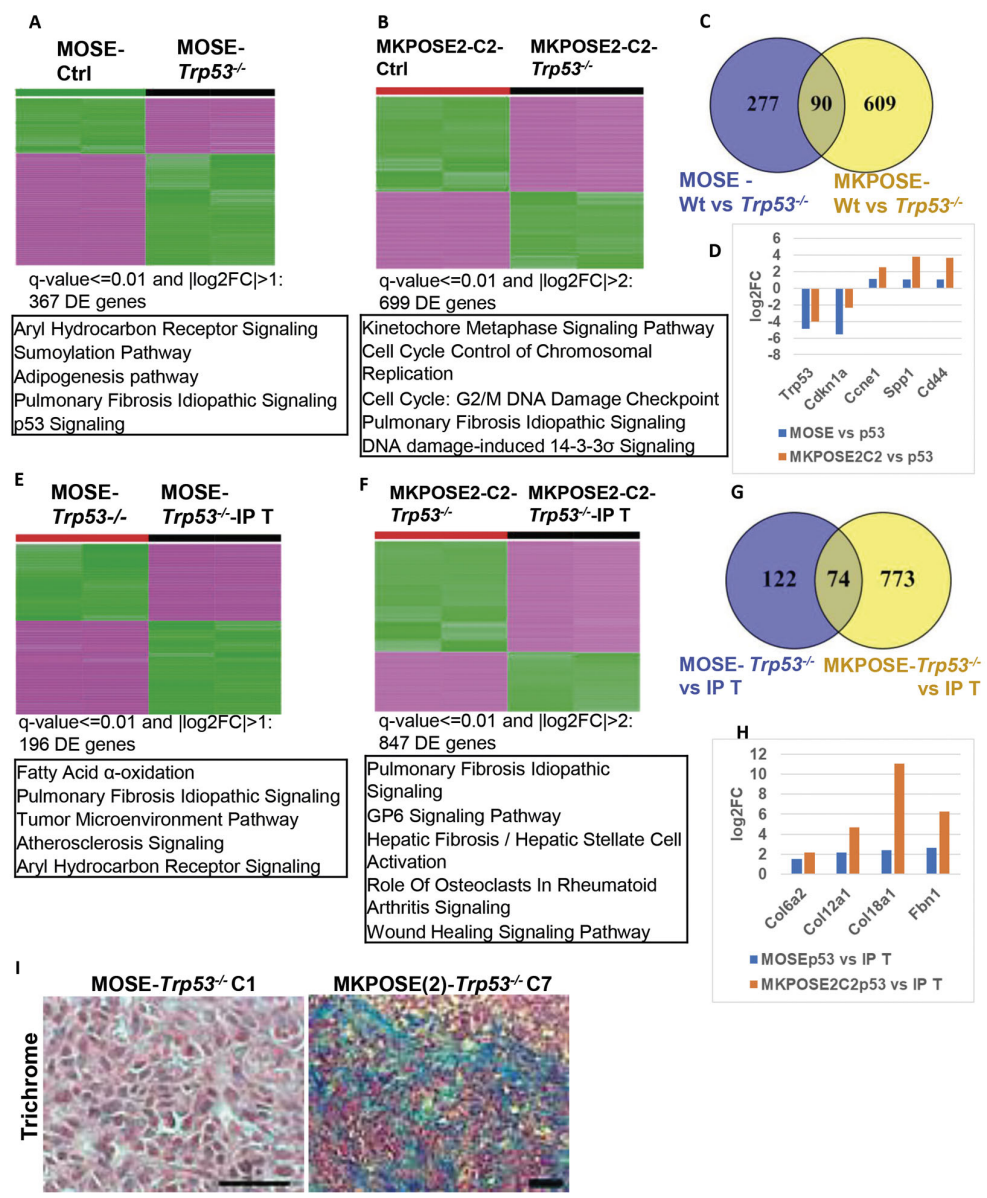
**Gene expression changes after Tp53 deletion and after *in vivo* passaging.** (A,B) Heatmap of differentially expressed (DE) genes (DESeq2) in MOSE-*Trp53*^−/−^ cells (A; q-value <0.01 and |log2FC|>1, *n*= 367 genes) and MKPOSE2-C2-*Trp53*^−/−^ cells (B; q-value <0.01 and |log2FC|>2, *n*=699 genes) compared to their respective, DNP-treated, controls. Top five pathways (by Ingenuity Pathway Analysis) are shown under each heatmap. Each cell line has two columns representing two biological replicates of same passage collected at two different time points, 5-6 h apart) on the same day. (C) Venn diagram of the DE genes shown in A (purple) and B (yellow). (D) Examples of five different genes from the Venn diagram intersection shown in C. Negative- and positive-mean value genes were downregulated or upregulated, respectively, after Trp53 deletion in MOSE (blue) or MKPOSE2 C2 cells (orange), compared to parental (wild-type, control) cells. *y*-axis, log2FC. (E,F) Heatmaps of DE genes in cell lines after *in vivo* passaging of MOSE-*Trp53*^−/−^ IP tumor nodule (E; q-value <0.01, |log2FC|>1, *n*=196 genes) and MKPOSE2-C2-*Trp53*^−/−^ IP tumor nodule (F; q-value <0.01, |log2FC|>2, *n*=847 genes). Comparisons use the before *in vivo* passaging cells as baseline. Number of replicates as described in A and B. (G) Venn diagram of genes used for heatmaps in E (purple) and F (yellow). (H) Examples of four genes commonly upregulated after *in vivo* passaging (intersection in G). (I). Trichrome stain visualizes collagen (blue), nuclei (dark brown) and cytoplasm (pink). One representative example (from more than five mice per model) is shown for each of the indicated models. Scale bars: 50 µm.

To fully characterize these two new high-grade serous ovarian cancer mouse models, we analyzed the RNAseq profiles of MOSE and MKPOSE2-C2 cells before and after *Trp53* deletion, and before and after *in vivo* passaging. Deletion of *Trp53* in MOSE and MKPOSE2-C2 cells triggers 367 and 699 differentially expressed (DE) genes, respectively (Fig. 4A,B; Table S1). In line with the *Trp53* null genotype confirmed above, expression of *Trp53* and its target *Cdkn1a* (*P21*) are among the most significantly downregulated in both MOSE-*Trp53^−/−^* and MKPOSE2-C2-*Trp53^−/−^* cells compared to their parental, *Trp53* wild-type counterparts (Fig. 4D; Table S1). As expected, the top five canonical pathways [Ingenuity Pathway Analysis (IPA)] are centered on *Trp53* signaling and cell cycle control. We note, however, that although an identical gene-editing protocol was applied to both, only a relatively small subset (*n*=90 genes) of the DE genes were commonly triggered by *Trp53* deletion in both cell populations (Fig. 4C; Table S1). However, among the overlapped DE genes, we identified upregulation of genes encoding several markers typically overexpressed in ovarian cancer: cyclin E1 (*Ccne1*) (Karst et al., 2014), RAD51-associated protein 1 (*Rad51ap1*) (Filipe et al., 2022), osteopontin (*Spp1*) (Kim et al., 2002) and its signaling receptor, *Cd44* (Sliutz et al., 1995) (Fig. 4D).

*In vivo* growth of MOSE-*Trp53^−/−^* and MKPOSE2-C2 -*Trp53^−/−^* cells resulted in changes in 196 and 847 genes, respectively (Fig. 4E,F; Table S2). The top canonical pathways point to tumor microenvironment and extracellular matrix remodeling, supporting the conclusion that these pathways contribute to the more rapid *in vivo* growth observed after *in vivo* passaging. Most notably, we note genes encoding collagens (*Col6a2*, *Col12a1*, *Col18a1*) and fibrillin 1 (*Fbn1*), which were commonly upregulated in cells after *in vivo* passaging, especially in MKPOSE2-C2-*Trp53^−/−^* cells (Fig. 4G,H; Table S2).These genes, often expressed in the stroma, can also be induced in epithelial tumor cells, especially under EMT or in aggressive, invasive states (Hong et al., 2023; Jiang et al., 2019; Zhang et al., 2023). Trichrome stain, used to visualize connective tissue, confirmed that of the two HGSOC models, tissue collagen deposition is more pronounced in the MKPOSE2-C2-*Trp53^−/−^* tumors (Fig. 4I). We also note that the aryl hydrocarbon and pulmonary fibrosis idiopathic signaling pathways are among the top dysregulated pathways (Fig. 4A,B,E,F). The aryl hydrocarbon receptor (AHR) promotes tumor growth, metastasis, genomic instability and chemoresistance, augments immune evasion and has been associated with poorer patient outcome (Deuster et al., 2019; Fauteux et al., 2023; Griffith and Frankel, 2024; Therachiyil et al., 2022). Importantly, there is mutual regulation between TP53 and AHR, and both influence genes in cell cycle control (Nguyen et al., 2023). Although the idiopathic pulmonary fibrosis pathway has not been directly studied in ovarian cancer, there are several signaling mechanisms, including via dysregulated TGF-β, Wnt and PI3K/AKT, that are also implicated in ovarian cancer progression (Ballester et al., 2021). Together, results in Figs 2-4 demonstrate that deletion of *Trp53* in two different immortalized, OSE-derived cell lines triggers cellular transformation. Importantly, IP injection of either cell line leads to high-grade serous orthotopic tumors with 100% penetrance, and lines obtained after *in vivo* passaging trigger accelerated disease. RNAseq analyses identified the genes modified by *Trp53* deletion in these two models, as well as genes modified by *in vivo* growth.

### *Pten* deletion leads to metastatic ovarian tumors with high-grade endometrioid histology

**Fig. 5.**
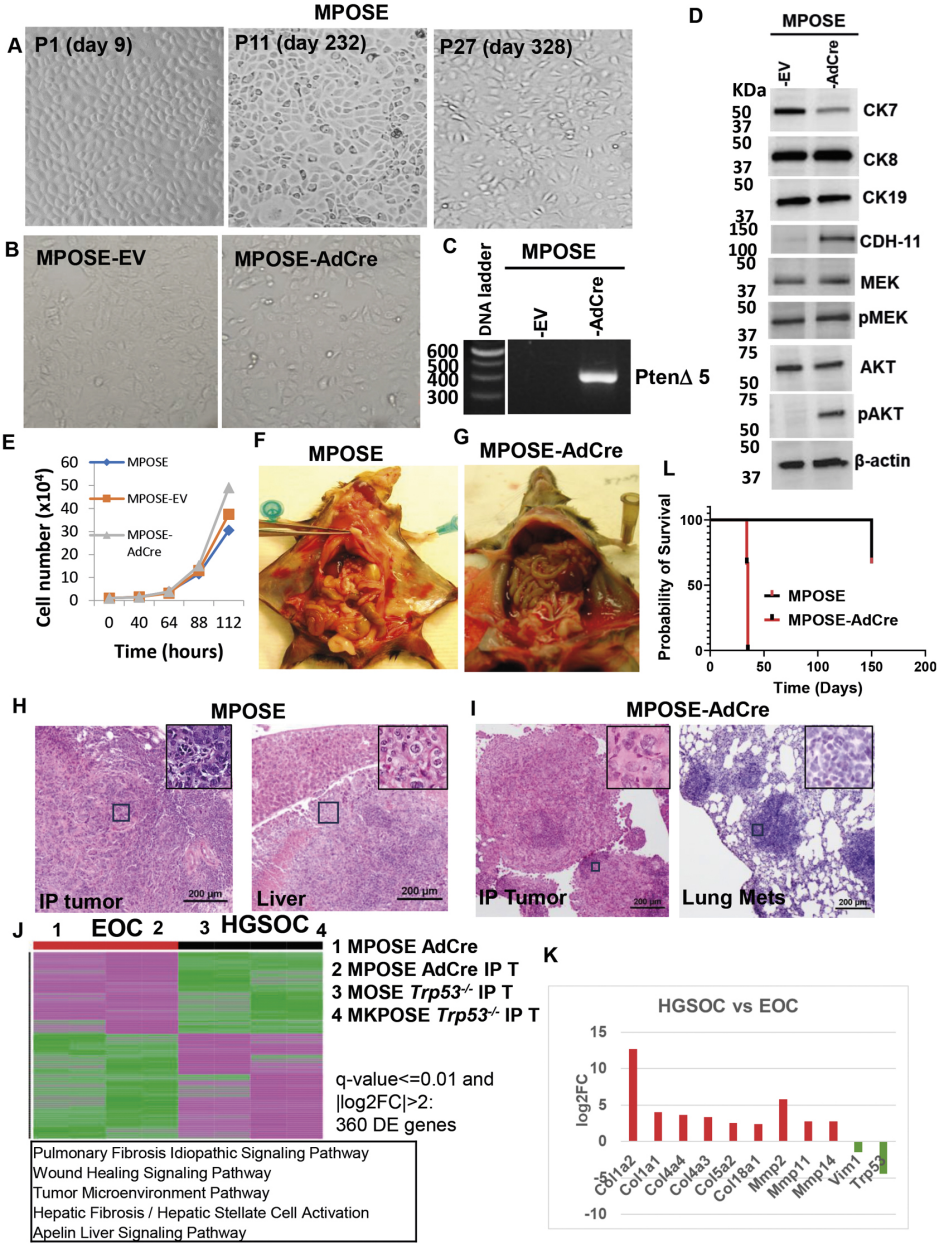
**Conditional deletion of Pten leads to aggressive ovarian tumors with endometrioid histology.** (A) Longitudinal changes in MPOSE cell morphology. (B) Upon immortalization, cells were exposed *in vitro* to Cre-encoding adenovirus (AdCre). Empty vector (EV) virus was used as control. (C) AdCre infection leads to homozygous deletion of *Pten* exon 5, resulting in a 420-bp PCR band. No band indicates wild type, which, in this case, is too long (>5 kb) to be amplified with a 1-min extension time. (D) Western blot of cell lysates obtained from MPOSE-AdCre or control (EV) cells blotted with antibodies specific for the proteins shown. β-actin was used a loading control. (E) *In vitro* cell growth curves. (F) Tumor growth after IP injection of 4 million MPOSE cells, which leads to peritoneal spread and ascites in one of three injected syngeneic mice. (G) IP injection of 2 million MPOSE-AdCre cells triggers peritoneal tumors, ascites and lung metastases in ∼5 weeks. (H) HE images of MPOSE tumors isolated from the indicated anatomical locations. The insets in the upper-right corner are from the fields indicated with black outline boxes. (I) HE of MPOSE-AdCre tumors isolated from different anatomical locations. The insets in the upper-right corner are from the fields indicated with black outline boxes. (J) Heatmap of DE genes (identified using DEseq2) comparing two endometrioid and two HGSOC models (q<0.01, |log2FC|>2, *n*=360 genes). The top five canonical pathways are listed under the heatmap. (K) Examples of nine genes that are upregulated and two genes that are downregulated in HGSOC compared to endometrioid tumors (red and green bars, respectively). *y*-axis, log2FC. (L) Survival curves of MPOSE and MPOSE-AdCre cell lines in syngeneic mice (three mice/group).

**Fig. 6.**
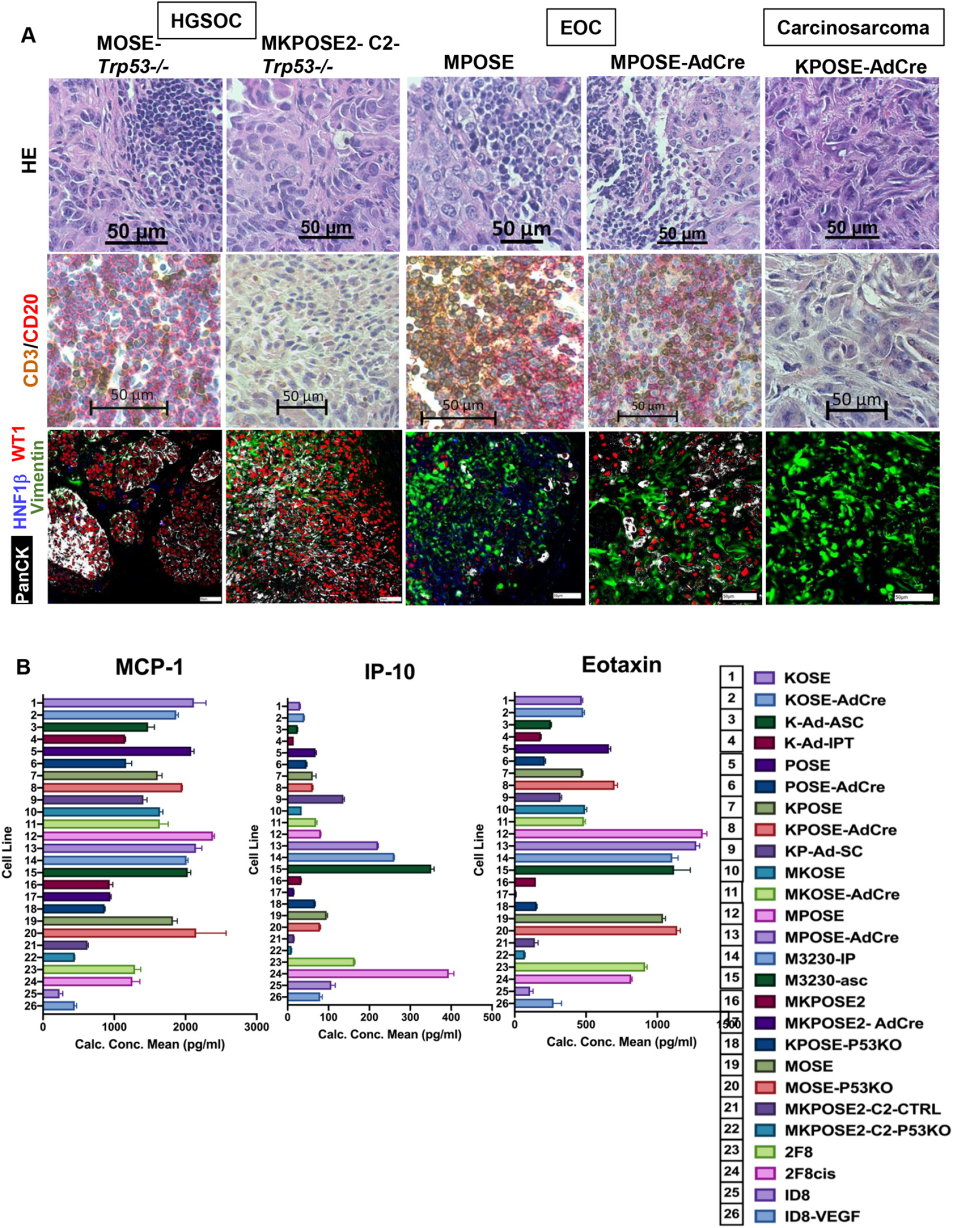
**Cell lines reproduce different ovarian cancer histotypes and have different chemokine secretion profiles.** (A) Syngeneic mice were injected IP with the specified cell lines. Tumors obtained at necropsy were fixed in formalin and paraffin embedded. Four-micron sections were used for HE staining (top row), dual CD3 (brown)/CD20 (red) IHC (middle row) and chip cytometry with four fluorescent markers (WT1, red; HNF1β, blue; pan-cytokeratin, white; vimentin, green) (bottom row). Scale bars: 50 µm. (B) Heterogeneity of secreted cytokine and chemokines across 29 murine cell lines. Murine IP-10 (CXCL10) and eotaxin were measured in cell culture supernatants by Meso Scale Discovery. MCP-1 was measured by enzyme-linked immunosorbent assay. Values represent average concentration (in pg/ml) of two technical replicates. Key is shown on the right. Additional measurements are shown in Fig. S8.

As with *Trp53*, we used the newly generated cell line MPOSE to model the deletion of tumor suppressor *Pten.* The MPOSE cell line was derived from the primary OSE cells harvested from the ovaries of MUC1^+/−^ Pten^loxP/loxP^ mice. Immortalized cells were established after 280 days of continuous *in vitro* culture (Fig. 5A). Exposure of late-passage MPOSE cells to AdCre *in vitro* did not significantly change the cell morphology (Fig. 5B) or *in vitro* proliferation curves (Fig. 5E). However, Cre-loxP recombination in MPOSE cells triggers homozygous loss of *Pten* exon 5 (responsible for phosphatase activity) (Dinulescu et al., 2005) (Fig. 5C) and increased pAKT expression (Fig. 5D). IP injection of MPOSE-AdCre [or control, empty vector (EV)-treated] parental cells led to extensive high-grade endometrioid tumors that develop throughout the abdomen, including the diaphragm, liver surface and peritoneal wall (Fig. 5F-I). The classification as ‘endometrioid’ has been prompted by the presence columnar, cytokeratin 7-expressing (Fig. 5D) epithelial cells and glandular structures, mild to moderate nuclear atypia and infrequent mitoses. The tumors also show absence of papillary structures, mucinous differentiation or clear cytoplasm, typically seen in serous, mucinous or clear cell carcinomas, respectively. Progression to terminal disease is significantly shorter for the MPOSE-AdCre model, with an average of 5 weeks (latency range 34-36 days, *n*=3 mice) after IP injection of 2×10^6^ cells, compared to parental MPOSE cells, the humane endpoints of which were reached more than 5 months post IP injection of 4×10^6^ cells, and only in one of three injected syngeneic mice, suggesting that this cell line is only partly transformed (Fig. 5L). Notably, mice receiving the cells exposed to AdCre (but not control, ‘empty’ adenoviral vector) also developed lung metastases, suggesting an increase in the *in vivo* metastatic potential post *Pten* deletion (Fig. 5I), a finding also supported by the low cytokeratin 7 and high cadherin 11 protein expression in this model (Fig. 5D) (Lin et al., 2022; Liu et al., 2023). Analysis of DE genes between the endometrioid and serous tumors identified fibrosis and collagen deposition as being upregulated in HGSOC (Fig. 5J,K; Table S3), whereas vimentin, a marker of cell invasion, is high in endometrioid tumors (Fig. 5K and Fig. 6A) (Usman et al., 2021). Together, these results suggest an invasive high-grade endometrioid ovarian cancer mouse model.

### Conditional activation of LSL-Kras^G12D^ and Pten^loxP/loxP^ deletion models carcinosarcoma

The KOSE, POSE and KPOSE cell lines (and their MUC1-expressing counterparts) originate in the OSE harvested from healthy ovaries of mice carrying silent conditional (Cre-loxP) mutations at the *Kras^G12D/+^* or *Pten^loxP/loxP^* loci, or both, respectively (Budiu et al., 2009, 2013; Dinulescu et al., 2005). The cells reached immortalization after 185, 187 and 162 days in culture, respectively, although they were not transformed, as assessed by soft agar colony formation. Activation of silent mutations via *in vitro* exposure to AdCre generates ‘daughter’ cell lines (KOSE-AdCre, POSE-AdCre and KPOSE-AdCre, respectively) characterized by increased signaling downstream of oncogenic *Kras^G12D^* (via MEK/ERK), downstream of AKT (via *Pten* deletion), or both (Table 1; Fig. S4). Unlike the parental cells, AdCre-treated cells are transformed and can trigger tumors *in vivo*. The KOSE-AdCre tumors show high-grade undifferentiated histology (Fig. S4) and are highly infiltrated by T and B cells that form peripheral node addressin (PNAd)-positive intratumoral lymphocytic conglomerates. In contrast, the KPOSE-AdCre cells are vimentin high and immune ‘cold’ and recreate carcinosarcoma histopathology (Fig. 6A; Fig. S4).

### Cell lines trigger tumors with different tumor antigen expression, immune infiltration profiles, and patterns of secreted cytokine and chemokines

MUC1 tumor-associated antigen is a current target of immune therapy in numerous adenocarcinoma types, including EOC (Chen et al., 2024). Some of our ovarian tumor models are derived from the OSE of MUC1 transgenic mice and express human MUC1 (Table 1). The two high-grade serous models, the MOSE-*Trp53^−/−^* model and MKPOSE2-C2-*Trp53^−/−^* model, have low and moderate MUC1 expression, respectively. The two endometrioid tumor models, MPOSE and MPOSE-AdCre, are MUC1 high (Fig. S5A). Transcriptionally, all 16 cell lines express various levels of other tumor-associated antigens, such as mesothelin (Hassan et al., 2016), survivin (Berinstein et al., 2015), folate receptor (Matulonis et al., 2023) and Erbb2 (Lafky et al., 2008) (Fig. S5B).

To define tumor inflammation, a key prognostic indicator and predictor to immune therapy responses (Kandalaft et al., 2022), we assessed the T and B tumor-infiltrating lymphocytes (TILs) in the five different tumor types described above, using dual CD3/CD20 (also known as MS4A1) immunohistochemistry (IHC). We observed high frequency of TILs and lymphocytic aggregates in the WT1-positive, cytokeratin-positive, MOSE-*Trp53^−/−^* HGSOC models (Fig. 6A). These aggregates showed positive staining for PNAd (Fig. S6), a high endothelial venule (HEV) marker (Weinstein and Storkus, 2016), suggesting that these intratumor aggregates are tertiary lymphoid structures (TLSs). The frequency of CD3-positive TILs was lower, and no B cells or TLSs were present, in the vimentin-high MKPOSE2-C2-*Trp53^−/−^* model (Fig. 6A).These profiles are further supported by the fact that inflammatory response gene signature-associated genes – such as *Il18*, *Il1r1*, *Lrf5* and *Vegfa* – show higher expression level in MOSE-*Trp53^−/−^* cells than in MKPOSE2-C2-*Trp53^−/−^* cells (Fig. S7).

Similar CD3/CD20 and PNAd expression pattern was also observed in the two endometrioid models (MPOSE and MPOSE-AdCre) (Fig. 6A). The KPOSE-AdCre carcinosarcoma model is WT1 and HNF1β negative, vimentin high, and shows low T-cell infiltration and no B cells or TLSs (Fig. 6A).

To further characterize the ability of these newly derived cells to spontaneously secrete chemokines and cytokines that can shape up the immune tumor microenvironment (TME), we interrogated the cell culture supernatants of 25 cell lines, for seven different secreted markers (IP-10/CXCL10, RANTES/CCL5, MCP-1/CCL2, eotaxin/CCL11, MIP2/CXCL2, TNFα/TNF and VEGF), using Meso Scale Discovery (MSD) multiplex technology (Fig. 6B; Fig. S8A). Selection of these markers was based on their broad roles in tumor–lymphoid and –myeloid immune cell interactions. As reference, we used the well-established and frequently employed, OSE-derived ID8 (Roby et al., 2000) and VEGF-transfected (ID8-VEGF) cells (Janat-Amsbury et al., 2006), as well as our previously reported tumor tissue-derived 2F8 and 2F8cis cell lines (Grabosch et al., 2019; Mony et al., 2015).

Compared to ID8 cells, all cells reported here secrete higher levels of MCP-1 (CCL2), which has wide range of chemoattracting properties for immune cells across several innate and adaptive immune cell lineages (Gschwandtner et al., 2019; Singh et al., 2021). Eotaxin (CCL11), an essential eosinophil chemoattractant (Ghaffari and Rezaei, 2023), was detected in all samples, and was abundantly secreted by most cells, similarly to MIP-2 (CXCL2), a chemokine important for activation of neutrophils (Charo and Ransohoff, 2006). We also detected the presence of T-cell chemokines IP-10 (CXCL10) and RANTES (CCL5), the latter with a more discrete expression pattern (Fig. 6B; Fig. S8). The 2F8cis cells, which trigger carcinosarcoma tumors that are abundantly infiltrated by T cells (Grabosch et al., 2019), express the highest levels of both RANTES and IP-10, likely explaining their immune ‘hot’ phenotype and partial response to immune checkpoint blockade (Grabosch et al., 2019). VEGF, a mediator of disease pathogenesis and a therapeutic target in ovarian cancer (Monk et al., 2016), was also detected, with some cells spontaneously secreting VEGF at levels close to those seen in overexpressing ID8 cells transfected with a VEGF-encoding plasmid [ID8-VEGF, (Janat-Amsbury et al., 2006)], included here as positive controls.

Several positive correlations were observed between some of these markers, across all the tested cells lines (Fig. S8B). MCP1 secretion shows positive correlation with eotaxin (correlation coefficient, 0.75), while some of the high MIP2-expressing cells also express high levels of TNFα (correlation coefficient, 0.65; Fig. S8B). A moderate positive correlation (correlation coefficient, 0.5; Fig. S8B) was also observed between RANTES and IP-10. In conjunction with the tumor immune infiltration profile, these cytokine secretion measurements further detail the diverse inflammatory profiles of these new tumor models.

## DISCUSSION

We report here a collection of novel murine ovarian cancer cell lines with distinct genotypic and phenotypic characteristics. Starting with primary OSE cells and following continuous *in vitro* propagation protocol, spontaneous immortalization was reached after 8-10 months in culture. If not spontaneously achieved during the prolonged *in vitro* growth, cellular transformation was obtained via subsequent genetic modifications of oncogenes or tumor suppressors, such as *Trp53* deletion, *Kras^G12D^* activation or *Pten* exon 5 deletion. All the resulting cell lines have been stably maintained and can be easily propagated in regular cell culture medium.

Through spontaneous metaplasia, dysplasia and transformation, the OSE (a type of mesothelium) can give rise to ovarian carcinomas resembling the morphology of the Müllerian epithelium. Using OSE cells to generate ovarian cancer cell lines was first reported more than two decades ago, and the OSE-derived ID8 cell line has been widely used to model ovarian cancer in syngeneic mice (Roby et al., 2000). Cook et al. performed transcriptomic profiling of several established HGSOC models for C57Bl/6 mice (Ballester et al., 2021). The expanded collection of cell lines described here further enhances the available preclinical research toolkit and offers several key advantages over the existing *in vivo* ovarian cancer models. Notably, these cell lines can also be adapted for use in alternative genetic backgrounds, such as beige mice, model more than one histotype and reproduce the biology of a human tumor antigen (MUC1), broadening their applicability.

Ovarian cancer is a heterogeneous disease that comprises different disease subtypes. High-grade serous ovarian tumors, the most frequent histotype, are almost entirely marked by variants in the *TP53* gene, leading to impaired P53 function (Zhang et al., 2016b). Orthotopic *in vivo* modeling of *de novo* HGSOC is challenging and requires several concomitant genetic modifications (Perets et al., 2013; Sherman-Baust et al., 2014; Zhai et al., 2017). Using two independent cell lines, we show here that a single genetic manipulation, resulting in *Trp53* deletion in the OSE, consistently promoted cell transformation and rendered cells capable of reproducing HGSOC *in vivo*, in immune-competent mice. These two new, WT1-positive, cytokeratin-positive, transplantable tumor models (MOSE-*Trp53^−/−^* and MKPOSE2-C2-*Trp53^−/−^*) mirror several important characteristics seen in human HGSOC, including peritoneal spread, ascites accumulation and intratumor immune infiltration, offering new opportunities for research into this ovarian cancer subtype. Although both models display the same HGSOC histology, they exhibit differential gene expression and immune profiles, with each model having distinct properties related to collagen secretion, stromal desmoplasia, immune cell infiltration, TLS formation and inflammatory cytokine secretion. The observed differences in gene expression among the cell lines, despite their shared histological tumor type *in vivo*, likely arise from clonal variability introduced during the immortalization process and microenvironment-independent adaptation *in vitro*. Each cell line may have acquired distinct epigenetic alterations, subtle genetic mutations, or differences in transcriptional regulatory networks during or after immortalization, which are not sufficient to alter tumor histotype but do influence gene expression profiles.

Our models include cell lines with silent, floxed mutations in *Kras^G12D^*, *Pten* or both. Although all loxP-carrying lines underwent spontaneous immortalization without AdCre, only MPOSE cells showed spontaneous transformation, forming tumors in one of three mice. This differential tumorigenicity may result from low-frequency, ‘leaky’ loxP recombination, which can occur without Cre and may escape PCR detection (Andrusaite and Milling, 2020; Song and Palmiter, 2018). Notably, MPOSE cells express significantly higher levels of *Ereg*, which binds to and activates EGFR and Erbb4 signaling to promote proliferation, survival, metastasis (Cheng et al., 2021; Sunaga et al., 2024) and angiogenesis (Gupta et al., 2007). Upregulation of other cancer-associated genes in MPOSE – such as *Ccnd1*, *Btc*, *Ankrd1*, *Oraov1*, *Cd44* and *Ifitm3* – as well as possible DNA mutations or fusions, may also contribute to tumorigenic differences and warrant further investigation. Activation of the silent Kras and Pten mutations (more often seen in non-serous tumors) can be achieved *in vitro* via Cre-loxP recombination post infection with AdCre. The AdCre and control EV-treated cells provide useful (‘paired’) substrates for studies on MEK/ERK and AKT tyrosine kinase pathway activity and for potential development of drugs targeting these oncogenic pathways (Kim et al., 2020). Importantly, we report that homozygous *Pten* deletion in MPOSE-AdCre cells triggers rapidly progressing, metastatic ovarian tumors consistent with the endometrioid histology, providing unique opportunities for *in vivo* modeling of this – less frequent – ovarian cancer histotype, for which very few syngeneic models currently exist.

Our data also provide insight into the capacity of these cell lines to spontaneously release chemokines and cytokines with important roles in chemoattraction of major immune cell types of both innate and adaptive lineages. The level of intratumor T-cell infiltration predicts response to immune therapies, including via immune checkpoint inhibitors, and choosing models with varying levels of T-cell infiltration will better reproduce disease heterogeneity seen in patients. Both IP-10/CXCL10 and RANTES/CCL5 have T-cell chemotactic properties and are abundantly expressed by some of these cell lines, including the 2F8cis cells, which have a highly T-cell inflamed (‘hot’) phenotype and respond to immune checkpoint blockade alone or in combination with cisplatin-based chemotherapy (Cascio et al., 2021; Grabosch et al., 2019; Mony et al., 2015). In line with this, our data from a Phase I clinical trial testing a novel chemo-immunotherapy combination show that increased loco-regional CXCL10 secretion serves as a hallmark of type 1, (interferon-induced) anti-tumor immune response (Orr et al., 2022), with beneficial roles in the immune TME (Li et al., 2021; Reschke et al., 2021). We also report that some of our models exhibit intratumor formation of T and B lymphoid aggregates (MOSE-*Trp53*^−/−^, MPOSE-AdCre) or more mature, PNAd-positive TLS-like formation (KOSE-AdCre). There is currently increased focus on the predictive or prognostic values of such findings in cancer, and these cell lines offer new venues of TLS exploration *in vivo* (Schumacher and Thommen, 2022; Xu et al., 2024).

We have previously reported that ovarian cancer patients with platinum-refractory disease, who were treated with IP IL-2 and responded favorably, had markedly increased eosinophils and serum CXCL11/eotaxin at the completion of treatment. Although the roles of eotaxin in ovarian cancer have not been extensively studied, it is known that eotaxin induction increases CD8-positive T cell recruitment to the TME in non-small lung cancer (Gao et al., 2019), promotes antitumor immunity and is associated with increased survival in colorectal cancer (Cao et al., 2021). The cell lines reported here secrete CXCL11 and could be used for further preclinical explorations of CXCL11/eotaxin-mediated immune regulation of the EOC immune microenvironment.

We acknowledge several limitations of our study. In humans, the proposed originating site for HGSOC cases is the fallopian tube epithelium, whereas evidence from genetically engineered mice supports both the fallopian tube and OSE as potential origins (Howitt et al., 2015; Zhang et al., 2019). Owing to the extended time required to immortalize primary oviduct cells, we used mouse primary OSE as the starting material in this study. Although the collection of cells reported here harbor key genetic alterations (such as deletions in *Trp53*, *Pten* and mutations in *Kras*), they do not reproduce some of the other important genetic features, such as BRCA1/2 biology and homologous recombination defects seen in HGSOC. Nevertheless, these models are highly versatile and amenable to further genetic manipulations to target *Brca1* or *Brca2*, as previously reported in ID8 cells (Walton et al., 2016, 2017). Some additional limitations come from the limited number of RNAseq technical replicates and from the fact that we performed RNA-based CNV inference, which is not a substitute for DNA-based approaches owing to biases, noise and incomplete genomic coverage (Barinka et al., 2022).

In summary, we present a versatile collection of immortalized murine cell lines with defined genetic traits, available in single, double or triple genetic combinations. These cell lines help in the selection of models for specific experimental objectives and reproduce several genotypic and phenotypic traits consistent with the HGSOC, carcinosarcoma or endometrioid ovarian cancer, for which few preclinical models currently exist. These biological tools can help model different immune TME profiles, providing new opportunities to advance preclinical research in immune-competent hosts.

## MATERIALS AND METHODS

### Experimental model details

#### Transgenic mice

All animal experiments were performed according to the protocol approved by University of Pittsburgh International Animal Care and Use Committee (IACUC). The mouse genetic backgrounds and breeding protocols have been previously described (Budiu et al., 2009; Dinulescu et al., 2005). The mice carrying LSL-*Kras^G12D/+^* and *Pten^loxP/loxP^* are on 129 S4/SvJae (H-2^b^) background. The *MUC1^+/−^* transgenic (*MUC1*.Tg) mice, originally on the C57BL/6J (also of H-2^b^) background (Peat et al., 1992), were first crossed with the LSL-*Kras^G12D/+^*mice. The F1 agouti littermates were backcrossed to mice on 129 S4/SvJae background, for at least ten generations, at which point it is predicted that 99.9% of the genome will match the 129S4/SvJae genome (Bouabe and Okkenhaug, 2013). Therefore, the haplotype of the fully syngeneic hosts for all described cell lines is H-2^b^.

Mouse colonies were maintained at Magee-Womens Research Institute animal facility, and genotyping was performed as previously reported (Budiu et al., 2009, 2013; Dinulescu et al., 2005). Given the focus on ovarian cancer, only female mice have been used in this study.

#### Generation of cell lines from primary OSE

For each cell line, ovaries from three to five female (6- to 8-week-old) mice were collected at necropsy. Periovarian fat was removed, and ovaries with intact surface were processed as previously reported, with slight modifications (Roby et al., 2000). Ovaries were gently placed in 10 ml TrypLE™ Express (Gibco) and incubated at 37°C, 5% CO_2_ for 30 min, with no shaking. Without touching the ovaries, the OSE cell-containing medium was collected, mixed with 5 ml complete DMEM (cDMEM) [DMEM (Corning) containing 10% heat-inactivated FBS (Corning), 100,000 U/l penicillin, 100 mg/l streptomycin, 2 mM L-glutamine, 1% non-essential amino-acids, 1 mM sodium pyruvate, 0.1 mM 2-mercaptoethanol (all from Sigma-Aldrich)] and centrifuged at 300 ***g*** at room temperature for 10 min. Pelleted cells obtained after OSE trypsinization were resuspended in 5 ml cDMEM supplemented with mouse EGF (2 ng/ml, Invitrogen) and 1× Insulin-Transferrin-Selenium (Gibco), and placed in a T25 flask (Corning). Primary OSE cells were propagated in a cell culture incubator, under sterile, standard conditions (37°C, 5% CO_2_). When reaching 80% confluency, cells were trypsinized with TrypLE™ Express (Gibco) and replated in cDMEM plus growth supplements. After the first trypsinization (passage 1), the FBS concentration in the culture medium was reduced to 4%. Growth supplements were removed from the medium (on average) at passage 16, which corresponded to time of immortalization. Time from primary OSE isolation to immortalization was between 8 and 10 months. Cell lines 2F8, 2F8cis, ID8 and ID8-VEGF (Zhang et al., 2002) were grown in cDMEM and used as reference, as indicated.

#### CRISPR/Cas9 editing of *Trp53*

In a six-well tissue culture plate, 2×10^5^ cells were seeded into a well in 3 ml antibiotic-free cDMEM and grown overnight to achieve 50-80% confluency at the day of transfection. For solution A, 3 µg mouse *Trp53* DNP or control DNP (Santa Cruz Biotechnology) was diluted into plasmid transfection medium (Santa Cruz Biotechnology) to a final volume of 150 µl, mixed well and allowed to stand for 5 min at room temperature. For solution B, 10 μl UltraCruz™ Transfection Reagent (Santa Cruz Biotechnology) was mixed with plasmid transfection medium to a final volume of 150 µl, mixed well and allowed to stand for 5 min at room temperature. Solution A was added dropwise to Solution B, mixed well and incubated for 20 min at room temperature. Culture medium in the six-well plate was replaced with fresh antibiotic-free cDMEM. The solution A+B (300 µl) mixture was added dropwise to the wells, mixed well, and incubated with the cells for 24 h at 37°C, 5% CO_2_. The GFP-positive transfected cells were sorted using an FACSAria™ Fusion sorter (BD Biosciences) followed by puromycin (Santa Cruz Biotechnology; 1 µg/ml for MOSE, 5 µg/ml for MKPOSE2, in cDMEM) selection. Surviving cells were resuspended in cDMEM at 50 cells/10 ml and added 100 µl/well to 96-well plates (Corning) to select single clones. Polymerase chain reaction (PCR) was used to validate the knockout of the *Trp53* gene with the following primers and conditions: forward primer 1, 5′-TCCGTTCTCTCTCCTCTCTTC-3′; reverse primer 1, 5′-TGTTGAGGGCTTACCATCAC-3′; 95°C, 5 min; 95°C, 30 s; 60°C, 40 s; 72°C, 1 min for 38 cycles; 72°C, 5 min. DNA fragments from individual clones were amplified using PCR with the same conditions shown above and the following primers: forward primer 2, 5′-CCTTGACACCTGATCGTTACTC-3′; reverse primer 2, 5′-ATTTCCTTCCACCCGGATAAG-3′. PCR fragments were purified using a QIAEX II Gel Extraction Kit (Qiagen) and sent to the Genomics Research Core at University of Pittsburgh for DNA sequencing using forward primer 1 as sequencing primer. A T100 Thermal Cycler (Bio-Rad) was used for PCR.

#### Conditional activation via AdCre infection

Cells were seeded in a 24-well plate (Corning) in 500 µl cDMEM without antibiotics and infected with Ad5CMVempty or Ad5CMVCre (University of Iowa Gene Transfer Vector Core) at a multiplicity of infection of 50. After overnight incubation at 37°C, 5% CO_2_, culture medium was discarded and replaced with 1 ml/well fresh cDMEM, and genomic DNA of the infected cells with at least five passages was isolated using an AllPrep DNA/RNA Micro Kit (Qiagen) and used as templates for PCR analysis to confirm *Pten* deletion or *Kras*^G12D^ activation. Primers were as follows: KrasF4, 5′-TGACACCAGCTTCCTATT-3′; KrasR4, 5′-GTAGCAGCTAATGGCTCTCAAAGG-3′; PtenF1, 5′-ACTCAAGGCAGGGATGAGC-3′; PtenR2, 5′-GCTTGATATCGAATTCCTGCAGC-3′. EmeraldAmp GT PCR master mix (TaKaRa) was used for PCR. Conditions for *Pten* were as follows: 94°C, 5 min; (94°C, 40 s; 62°C, 40 s; 72°C, 1 min) ×38; 72°C, 5 min. Conditions for *Kras* were as follows: 94°C, 5 min; (94°C, 40 s; 60°C, 40 s; 72°C, 1 min) ×38; 72°C, 5 min. When the *Pten* gene is intact, the length of the DNA between the *Pten* primers is >5 kb, and no visible PCR product will be produced with 1 min elongation time. After AdCre infection and successful Cre-*loxP* recombination, most of the region between the primers is removed, resulting in a PCR product of ∼400 bp.

#### Soft agar colony formation assay

Cells from different OSE cell lines were seeded into 24-well plates for the soft agar cell transformation detection assay (Millipore), using the manufacturer's protocol. Filtered H_2_O and cDMEM were used to prepare agar solutions. In brief, 24-well plates (Corning) were coated with 0.8% base agar solution, and 25 µl of 2.5×10^3^ cells was mixed with 975 µl 0.4% top agar solution and added to a 0.8% base agar-coated plate with 250 µl/well, in duplicates. The cells were cultured at 37°C, 5% CO_2_, for 21-28 days. Colony formation was assessed with an inverted microscope (Zeiss Invertoskop 40C).

#### *In vivo* tumor growth and sub-tumor cell line generation

A PCR Mycoplasma Detection Kit (TaKaRa) was used to rule out mycoplasma contamination. Cells (2-5×10^6^) from newly generated cell lines were washed and resuspended in sterile DPBS (Gibco) and injected intraperitoneally (200 µl) or subcutaneously (100 µl) into immune-competent, 6- to 8-week-old, syngeneic female hosts to check tumor growth in different microenvironments. Mice were monitored for at least 5 months post tumor challenge. Tumor-bearing mice were sacrificed when humane endpoints were reached, as per approved IACUC protocol, and as previously described (Budiu et al., 2013). Tumor nodules and ascites fluid were collected at necropsy. Lungs were also collected to check for lung metastases. Ascites cells and supernatants were separately cryopreserved until ready to use. Tumor nodules were cut into ∼8 mm^3^ pieces and cultured in cDMEM medium for ∼1-2 weeks, until tumor cells grew out from the tumor tissue and attached to the bottom of the flask. The remaining tumor tissue was then removed, and attached cells were trypsinized using TrypLE™ Express (Gibco) and subsequently passaged for at least ten passages before immortalization was obtained.

#### IHC and trichrome staining

Tumor nodules isolated at necropsy were placed in 10% formalin solution (Sigma-Aldrich) for 24-72 h, transferred to 70% ethanol for 48-72 h, paraffin embedded and sectioned. Four-micron sections were used for Hematoxylin and Eosin (HE) and trichrome staining at the Pathology Core of Magee-Womens Research Institute. IHC was performed using the following antibodies: anti-CD3 (Abcam, ab16669, 1:50), anti-CD8 (Cell Signaling Technology, 98941s, 1:100), anti-WT1 (Abcam, ab89901, 1:300), anti-Sma (Abcam, ab124964, 1:1000). anti-CD20 (Abcam, ab271288, 1:100) and anti-PNAd (BioLegend, 120801, 1:50). Dual staining for anti-CD3 (Abcam, Clone SP-7, 1:50) and anti-CD20 (Abcam, Clone GOT214A, 1:100) was performed using 4 µm formalin-fixed paraffin-embedded (FFPE) tissue sections. Tissue sections were hydrated using xylene and ethanol. Antigen retrieval was performed in Tris-EDTA (pH 9.0) buffer, in a water bath (95°C), for 20 min. Blocking was performed in 0.3% H_2_O_2_/methanol and 2% bovine serum albumin (BSA) for 20 min each. Dako EnVision+System-HRP Labelled Polymer Anti-Rabbit dropper and Goat Anti-Rat IgG Alkaline Phosphatase (1:100) were used as secondary antibodies. ImmPACT DAB EqV (1:1) and ImmPACT Vector Red were used as chromogens. Tissues were then counterstained with Hematoxylin and dehydrated.

A single-stain IHC protocol for PNAd (BioLegend, clone MECA-79, 1:50) used similar antigen retrieval and blocking steps as for the dual-stain protocol. eBioscience Goat Anti-Rat IgG HRP (1:250) was used as secondary antibody. ImmPACT DAB EqV (1:1) was used as chromogen, and Hematoxylin was used for counterstaining.

#### Western blotting

Cells lysates (20 µg) from cell lines were loaded onto 4-20% Mini-PROTEAN^®^ TGX™ Precast Gel (Bio-Rad), run at 160 V for 1 h and transferred at 120 mA for 2 h to nitrocellulose membranes (Bio-Rad). The following primary antibodies and corresponding dilutions were used to stain the membranes: anti-MEK1/2 (Cell Signaling Technology, D1A5, 1:2500), anti-pMEK1/2 (Ser217/221) (Cell Signaling Technology, 41G9, 1:2500), anti-AKT (pan) (Cell Signaling Technology, C67E7, 1:2500), anti-pAKT (Ser473) XP^®^ (Cell Signaling Technology, D9E, 1:2500), anti-β-actin (Sigma-Aldrich, A1978, 1:6000), anti-cytokeratin 7 [EPR17078] (Abcam, ab181598, 1:10000), anti-cytokeratin 8 [EP1628Y] (Abcam, ab53280, 1:20,000), anti-cytokeratin 19 [EPNCIR127B] (Abcam, ab133496, 1:20,000) and anti-cadherin 11 (Invitrogen, 717600, 1:2500). All primary antibodies were diluted in 2% BSA (Fraction V, Thermo Fisher Scientific) in PBS and were incubated with the membranes at 4°C overnight. Secondary horseradish peroxidase (HRP)-labeled goat-anti-mouse or goat-anti-rabbit IgG (Bio-Rad) were diluted 1:6000 in 2% BSA in PBS with 0.05% Tween 20 (Fisher Chemical) and incubated at room temperature for 1 h. SuperSignal™ West Femto Maximum Sensitivity Substrate (Thermo Fisher Scientific) was used to develop the membranes. Images were taken by a Chemidoc XRS (Bio-Rad).

#### MSD

Cells (1×10^6^) were resuspended in 1.5 ml cDMEM, plated in six-well plates and cultured for 15 h at 37°C, 5% CO_2_. Cell-free cell culture supernatants from 26 different cell lines were cryopreserved (−80°C) until ready to use. Aliquots were thawed and analyzed with the MSD platform, according to the manufacturer's protocol for U-PLEX Biomarker Group 1 (mouse) Multiplex Assays (MSD). The U-PLEX panel consisted of eotaxin (CCL11), IP-10 (CXCL10), MCP-1 (CCL2), MIP-2 (CXCL22), RANTES (CCL5), TNF-α, VEGF-A and 6CKine/CCL21. The samples were run in duplicates. The plates were scanned on the MSD MESO QuickPlex SQ 120 imager. Data were analyzed in Discovery Workbench 4.0 (MSD) and MSFT Excel.

#### Chip cytometry multiplexed imaging of tumors

Four-micron FFPE sections were placed on coverslips from Bruker Spatial Biology (formerly Canopy Biosciences) at Magee-Womens Research Institute pathology core. The tissue-mounted coverslips were incubated at 60°C for 2 h or overnight, and then put into xylene for 5 min, fresh xylene for 5 min, 100% ethanol (Fisher Scientific) for 5 min, fresh 100% ethanol for 5 min, 90% ethanol for 5 min, 70% ethanol for 5 min, 50% ethanol for 5 min and distilled H_2_O for 5 min, followed by boiling the coverslips in Tris-EDTA, pH 9 with 0.05% Tween 20 for 20 min, and 5 min incubation in PBS. The coverslips were mounted to Zellsafe FFPE tissue chips (Bruker Spatial Biology), and 1 ml storage buffer (Bruker Spatial Biology) per chip was added to the chips and stored at 4°C until further use. The following antibodies were used to stain the prepared tissue chips: anti-HNF1B polyclonal antibody (Proteintech, 12533-1-AP, 1:1500) with PE-donkey-anti-rabbit IgG (minimal x-reactivity; BioLegend, 406421, 1:300) as the secondary antibody, BUV395 mouse anti-human MUC1 (BD Biosciences, CD227, 1:300), Alexa Fluor^®^ 488 anti-vimentin (BioLegend, 677809, 1:600), Alexa Fluor^®^ 488 anti-WT1 (Abcam, ab202635, 1:200), PE-cytokeratin, pan antibody (Novus, C-11, 1:1500) and eFluor™ 570 anti-alpha-smooth muscle actin (eBioscience, 1A4, 1:600). A ZellScannerONE™ (Bruker Spatial Biology) with ZellScanApp and ZKWApp (version 19-08-2020) was used to scan and acquire images from the chips.

#### ELISA

MCP-1 ELISA (R&D Systems) was run according to the manual. A 96-well plate (IMMULON 4HBX, Thermo Fisher Scientific) was coated with capture antibody (200 ng/ml, 100 µl/well) overnight and blocked with reagent diluent (300 µl/well) for 2 h. Cell culture supernatants were 1:10 diluted in reagent diluent (no dilution for MKPOSE2-AdCre) and added 100 μl/well in duplicates, followed by detection antibody (50 ng/ml, 100 µl/well) and Streptavidin-HRP working solution (100 µl/well). Substrate solution (100 µl/well) was added and incubated for 20 min, after which stop solution (50 µl/well) was added. An Infinite 200 Pro plate reader (TECAN) was used to measure absorbance (optical density at 450 nm).

#### Gene expression and CNV analyses.

From each cell line, we collected two RNA samples using AllPrep^®^ DNA/RNA isolation kit (Qiagen), from two time points (5-6 h apart) of the same passage, representing two biological replicates. The samples were sent to Novogene for mRNA sequencing (RNAseq), and analyses were performed in house. Genes with relatively low expression levels were filtered out by ‘filterByExpr’ in the R package edgeR (Robinson et al., 2010). Expression of the remaining genes was normalized using the R packages DESeq2 (Love et al., 2014). DESeq2 was used to identify DE genes. Q-values were calculated via Benjamini–Hochberg correction (Benjamini and Hochberg, 1995) based on the *P*-values generated by DESeq2 for each gene. DE genes were jointly determined by their q-values (q≤0.01) and absolute values of logarithm of fold change (FC) (estimated by DESeq2 and denoted by |log2FC|; |log2FC|>1 for MOSE-*Trp53^−/−^* cells and MOSE-*Trp53^−/−^* IP tumor nodule; |log2FC|>2 for the others). IPA by QIAGEN used DE genes to identify significantly enriched pathways.

CNV calling was conducted using the R package CaSpER60 adopted for murine cell lines. Because CaSpER exempts the use of matching normal samples by only focusing on heterozygous single-nucleotide variant (SNV) candidates, which can be estimated from healthy samples, we used the ovary mRNA profiles of five normal mice from a comparison study found on Gene Expression Omnibus (GEO) under the accession number GSE196650. The reference genome used to sort SNVs and map the mutated loci to genes was GRCm38, and the centromere information used to annotate chromosome-level or arm-level CNV events was extracted from the same reference. The parameter values of the CaSpER algorithm [the numbers of smoothing scales of expression signal and allele-based frequency shift (BAF) signal, and the thresholding number of concordant pairs (the segments of expressional signal and BAF signal)] were kept as default.

## Supplementary Material

10.1242/dmm.052177_sup1 Supplementary information

Table S1. MOSE vs MOSE Trp53 null (n=367 DE genes, heatmap in Fig 4A)

Table S2. MOSETrp53nulll vs MOSETrp53null IP Tumor (n=196 DE genes, heatmap in Fig. 4E)

Table S3. Endometrioid versus HGSOC (n=360 DE genes, heatmap shown in Fig. 5J)
