# Supplementary material for: Murine cell lines with defined mutations model different histological subtypes of epithelial ovarian cancer
Source: Dis Model Mech. 2025 Jul 28;18(7):dmm052177. doi: 10.1242/dmm.052177 (PMC12352289; doi:10.1242/dmm.052177)
Supplement: Supplementary information [file dmm-18-052177-s1.pdf]

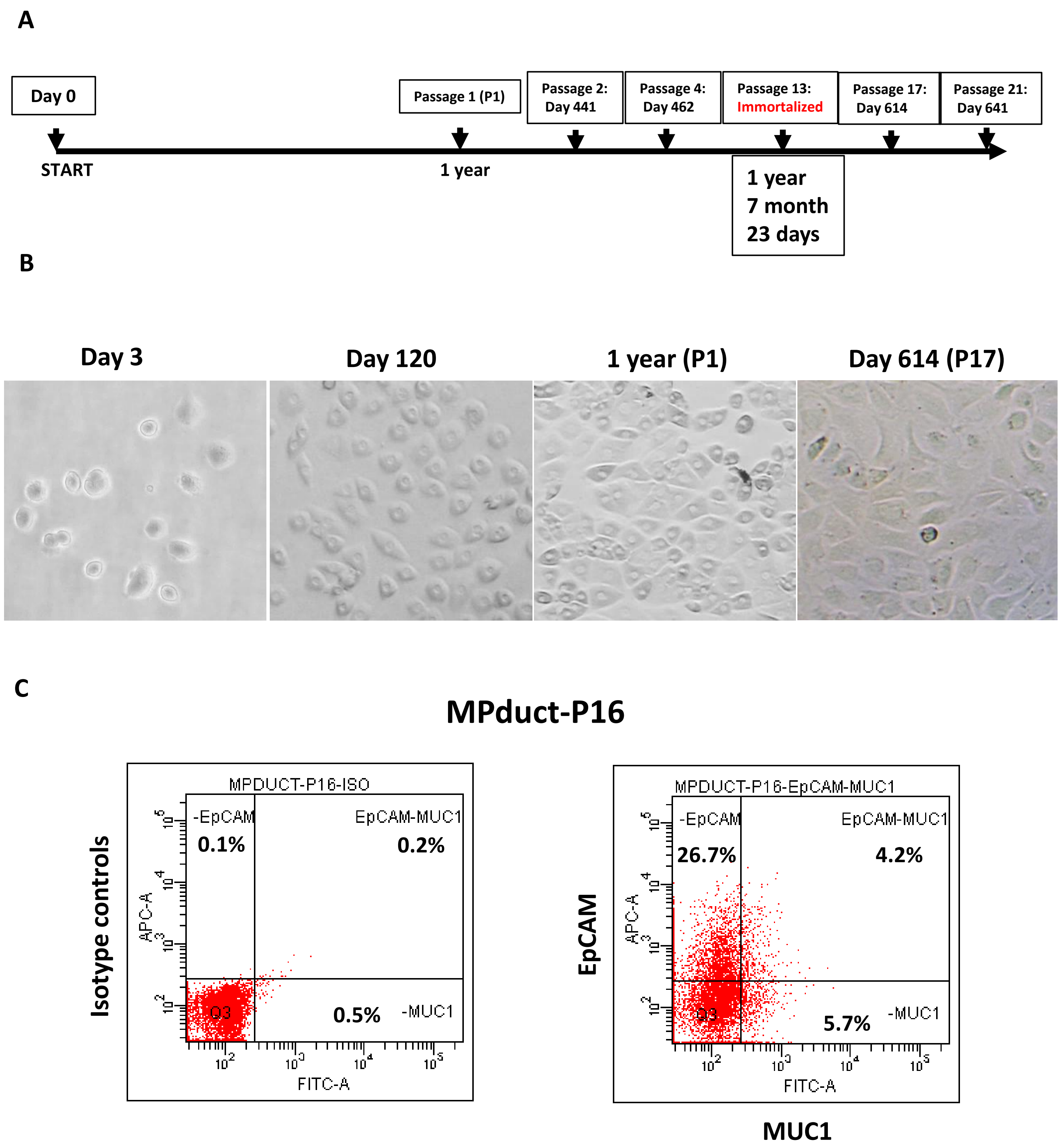

Fig. S1. Generation of cell lines from oviductal epithelial cells. (A) Oviducts were collected from five MUC1-Pten (MP) double transgenic mice and processed using the gentle trypsinization protocol used for OSE. The primary oviductal cells were cultured for extended time to generate a cell line (MPduct), as shown. (B). Morphology of different passages of cells in culture, at the indicated time points. (C). Flow cytometry measurement of percent MUC1 and EpCAM positive MPDuct cells at passage 16 (MPduct-P16). Gates were set using isotype control antibodies.

Disease Models & Mechanisms • Supplementary information

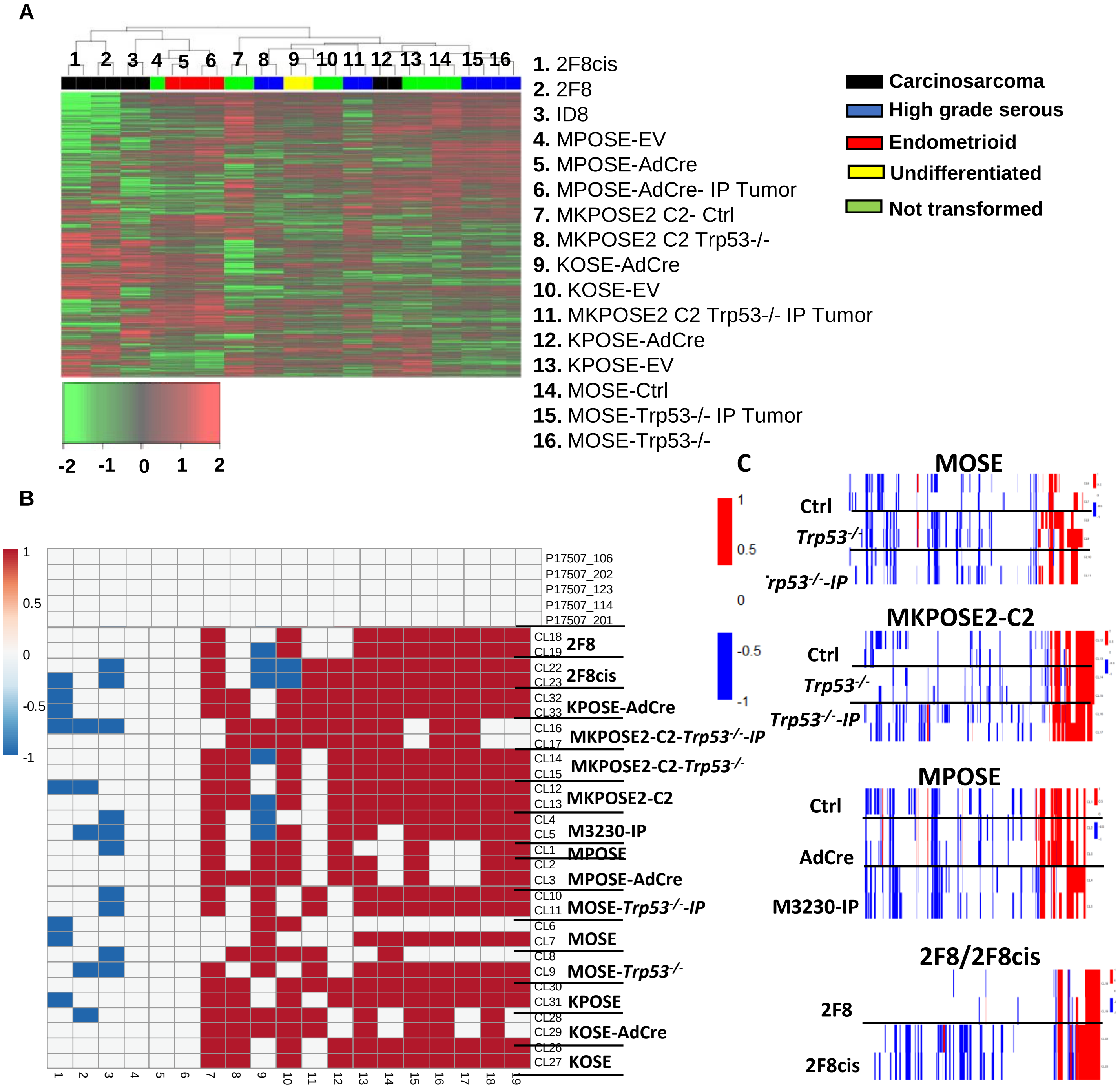

Fig. S2. **Copy number variations (CNVs) analyses using RNAseq data.** (A) Heatmap using normalized gene expression (n=12428 genes, R package DESeq2) across 16 different cell lines. Except for sample 4 (MPOSE-EV), which was run as single, all other cell lines were run as duplicates. Clustering across all 31 samples is shown on top. Color code represents different histologies. Green bar represents cells that are not transformed, i.e. do not form tumors in vivo. (B). Heatmap of copy number variations (CNV), inferred from RNAseq data. .X axis- Chromosome numbers. Y axis- cell line names. Top five rows represent normal ovaries, used as reference. Amplifications (red) and deletions (blue) are shown across all 19 chromosomes. Results from two technical replicates are shown, with the exception of MPOSE cells, which were included as a single sample. (C) Examples of CNV before and after Trp53 deletion and after in vivo passaging in MOSE and MKPOSE2-C2 cells (top 2 panels) after AdCre mediated Pten inactivation and in vivo passaging (MPOSE) and in 2F8/2F8cis cells (bottom panel). Black horizontal lines were added to aid with visualization between the technical replicates, listed from top to bottom. Amplification (red) and deletion (blue) are shown according to the scale at the top left (enlarged)

Disease Models & Mechanisms • Supplementary information

A

MOSE-*Trp53*$^{-/-}$-C1 1 MTAMEESQSDISLELPLSQETFSGLWKLLPPEDILPSPHCMDDLLLPQDVEEFFEGPSEA 60
Mouse *Trp53*-WT 1 MTAMEESQSDISLELPLSQETFSGLWKLLPPEDILPSPHCMDDLLLPQDVEEFFEGPSEA 60

MOSE-*Trp53*$^{-/-}$-C1 61 LRVSGAPAAQDPVTETPGPVAPAPATPWPLSSFVPSQKTYQGNYGFHLGFLQSGTAKSVM 120
Mouse *Trp53*-WT 61 LRVSGAPAAQDPVTETPGPVAPAPATPWPLSSFVPSQKTYQGNYGFHLGFLQSGTAKSVM 120

MOSE-*Trp53*$^{-/-}$-C1 121 CTYSPPLNKLFCQLAKTCPVQLWVSATPPAGSRVRAM**ES** 159
Mouse *Trp53*-WT 121 CTYSPPLNKLFCQLAKTCPVQLWVSATPPAGSRVRAMAIYKKSQHMTEVVRRCPHHERCS 180

Mouse *Trp53*-WT 181 DGDGLAPPQHLIRVEGNLYPEYLEDRQTFRHSVVVPYEPPEAGSEYTTIHYKYMCNSSCM 240
Mouse *Trp53*-WT 241 GGMNRRPILTIITLEDSSGNLLGRDSFEVRVCACPGRDRRTEEENFRKKEVLCPELPPGS 300
Mouse *Trp53*-WT 301 AKRALPTCTSASPPQKKKPLDGEYFTLKIRGRKRFEMFRELNEALELKDAHATEESGDSR 360
Mouse *Trp53*-WT 361 AHSSYLKTKKGQSTSRHKKTMVKKVGPDSD 390

B

MKPOSE2-C2-*Trp53*$^{-/-}$-C7 1 MTAMEESQSDISLELPLSQETFSGLWKLLPPEDILPSPHCMDDLLLPQDVEEFFEGPSEA 60
Mouse *Trp53*-WT 1 MTAMEESQSDISLELPLSQETFSGLWKLLPPEDILPSPHCMDDLLLPQDVEEFFEGPSEA 60

MKPOSE2-C2-*Trp53*$^{-/-}$-C7 61 LRVSGAPAAQDPVTETPGPVAPAPATPWPLSSFVPSQKTYQGNYGFHLGFLQSGTAKSVM 120
Mouse *Trp53*-WT 61 LRVSGAPAAQDPVTETPGPVAPAPATPWPLSSFVPSQKTYQGNYGFHLGFLQSGTAKSVM 120

MKPOSE2-C2-*Trp53*$^{-/-}$-C7 121 CTYSPPLNKLFCQLAKTCPVQLWVSATPPAGSRVRAMAIYKNDGGRETLPPP 172
Mouse *Trp53*-WT 121 CTYSPPLNKLFCQLAKTCPVQLWVSATPPAGSRVRAMAIYKKSQHMTEVVRRCPHHERCS 180

Mouse *Trp53*-WT 181 DGDGLAPPQHLIRVEGNLYPEYLEDRQTFRHSVVVPYEPPEAGSEYTTIHYKYMCNSSCM 240
Mouse *Trp53*-WT 241 GGMNRRPILTIITLEDSSGNLLGRDSFEVRVCACPGRDRRTEEENFRKKEVLCPELPPGS 300
Mouse *Trp53*-WT 301 AKRALPTCTSASPPQKKKPLDGEYFTLKIRGRKRFEMFRELNEALELKDAHATEESGDSR 360
Mouse *Trp53*-WT 361 AHSSYLKTKKGQSTSRHKKTMVKKVGPDSD 390

Fig. S3. DNA sequencing analyses of *Trp53* gene. (A). DNA sequencing confirms the functional deletion and predicts a truncated Trp53 protein of 159 amino acids (aa), compared to 390 aa in wild type in MOSE cells. Changes at the DNA repair site led to newly added aa residues (in red). (B). DNA sequencing confirms the homozygous Trp53 deletion and predicts a truncated protein (172 aa, compared to 390 aa in wild type) in MKPOSE2-C2 cells. Changes at the DNA repair site led to newly added aa residues (in red).

Disease Models & Mechanisms • Supplementary information

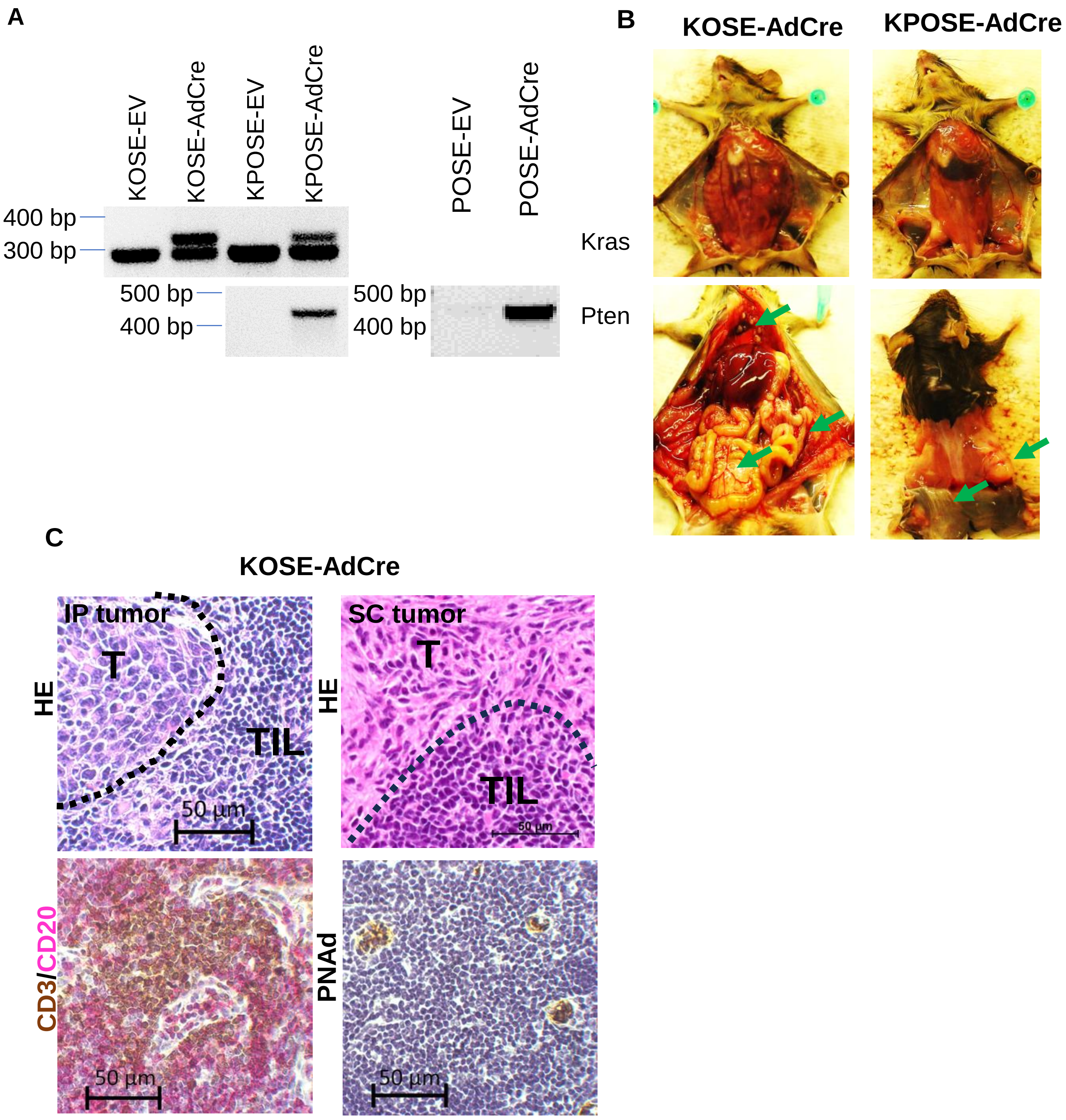

Fig. S4. In vitro exposure of OSE-derived immortalized cells to AdCre leads to Cre-loxP recombination at the KrasG12D, Pten or both loci. (A) KOSE, KPOSE, POSE cells were infected with empty vector (EV) or AdCre at the MOI of 50. PCR of genomic DNA confirmed the Cre-loxP recombination and activation of $Kras^{G12D}$ and/or deletion of Pten. (B) KOSE-AdCre or KPOSE-AdCre were injected IP. ($4x10^6$ cells) and SC ($2x10^6$ cells) into three syngeneic mice. After 4 weeks, all three KOSE-AdCre injected mice developed IP tumors (arrows) with hemorrhagic ascites and 2 out of three mice also grew SC tumors. All three KPOSE-AdCre injected mice developed SC. tumors, with no visible IP tumor or ascites. (C) Top panels- HE of IP (left) and SC (right) of KOSE-AdCre tumor nodules with high grade undifferentiated, carcinoma histology. T- tumor area; TIL- tumor infiltrating lymphocytes. Lower panels- (left) IHC staining of KOSE-AdCre IP tumor. Dual IHC stain for CD3 (brown) and CD20 (red) shows T and B conglomerates; (right)- IHC staining for peripheral node addressin (PNAd). Scale bar, 50um.

Disease Models & Mechanisms • Supplementary information

A

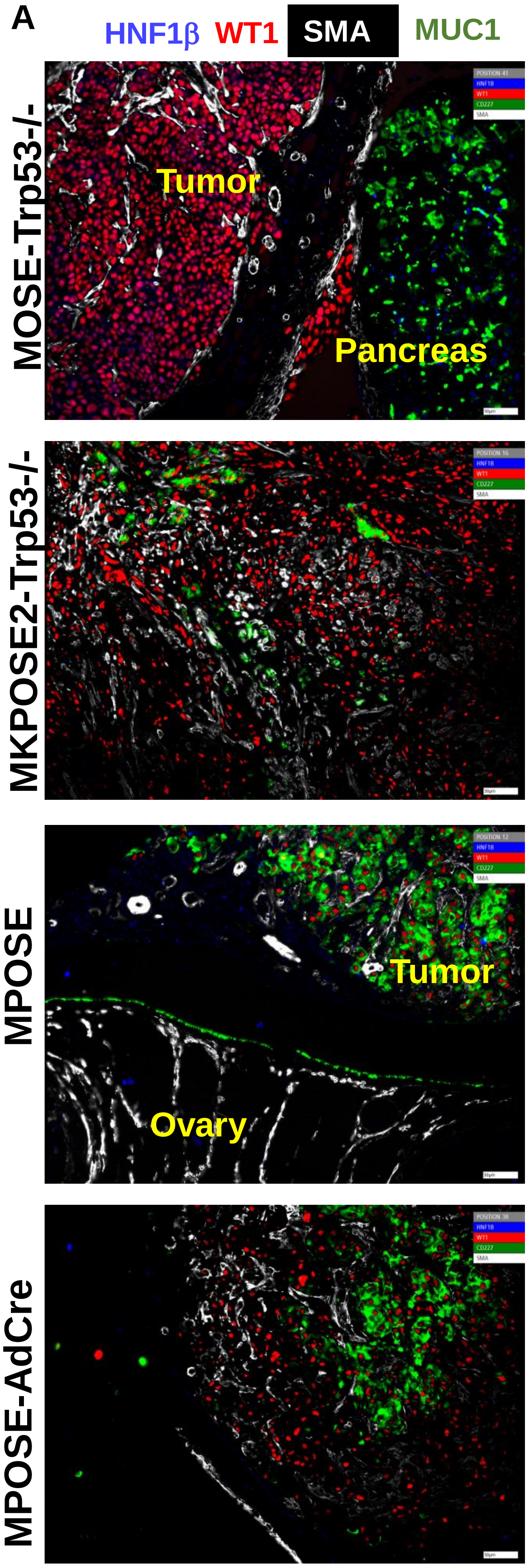

B

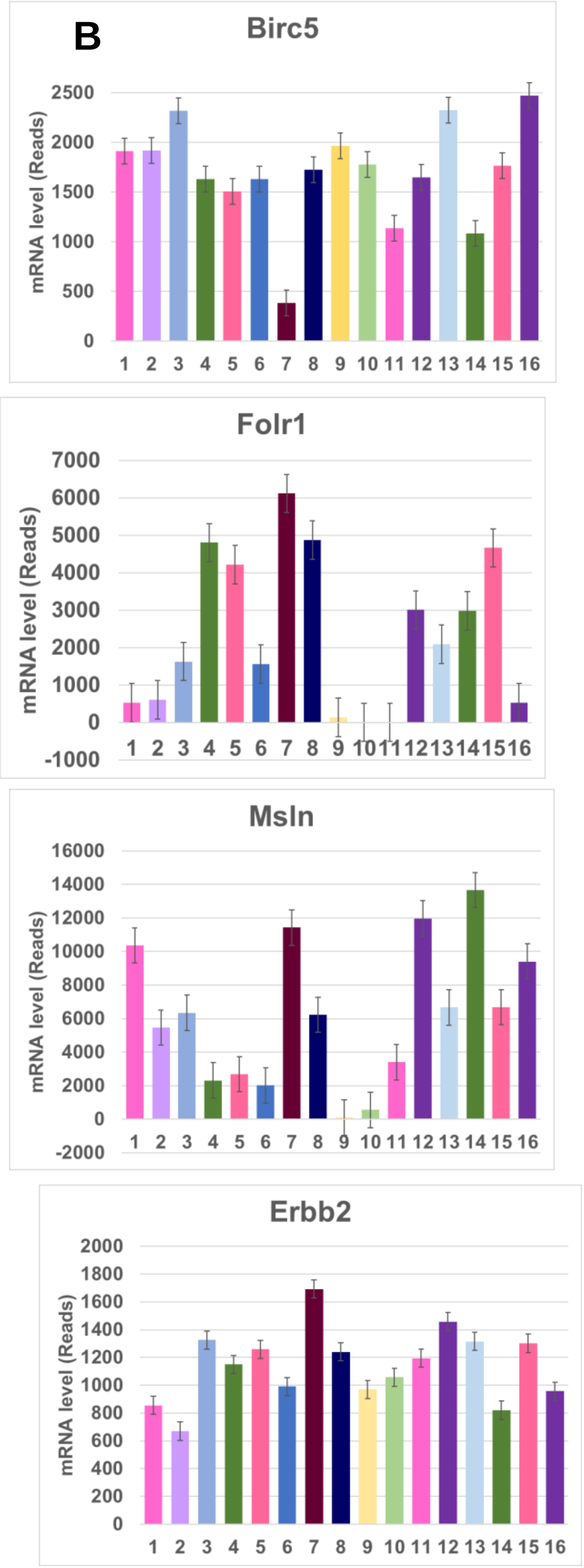

1. MPOSE
2. MPOSE-AdCre
3. MPOSE-AdCre-IP T
4. MOSE
5. MOSE- Trp53-/-
6. MPOSE—Trp53-/- IP T
7. MKPOSE2 C2
8. MKPOSE2 C2- Trp53-/-
9. MKPOSE2 C2 Trp53-/-- IP T
10. 2F8
11 2F8Cis
12. KOSE
13. KOSE-AdCre
14. KPOSE-
15. KPOSE-AdCre
16. ID8

Disease Models & Mechanisms • Supplementary information

Fig. S5. Tumor antigen expression across different cell lines. (A) Chip cytometry of four different tumors, using antibodies specific for MUC1 (green), HNF1β (blue), SMA (white) and WT1 (red). The tumors express various levels of human MUC1 antigen, (green). Except for MOSE-Trp53-/-, all other tumors are MUC1 positive. For each model, cells were injected into hosts that are transgenic for human MUC1 and show same MUC1 protein expression as in humans. This explains the positive green staining in pancreas (top panel) and on the OSE (third panel from top). (B) Expression levels (RNASeq) of genes encoding for tumor associated antigens survivin (Birc5), folate receptor alpha (Folr1), mesothelin (Msln) and Erbb2. Average of duplicate values are shown.

Disease Models & Mechanisms • Supplementary information

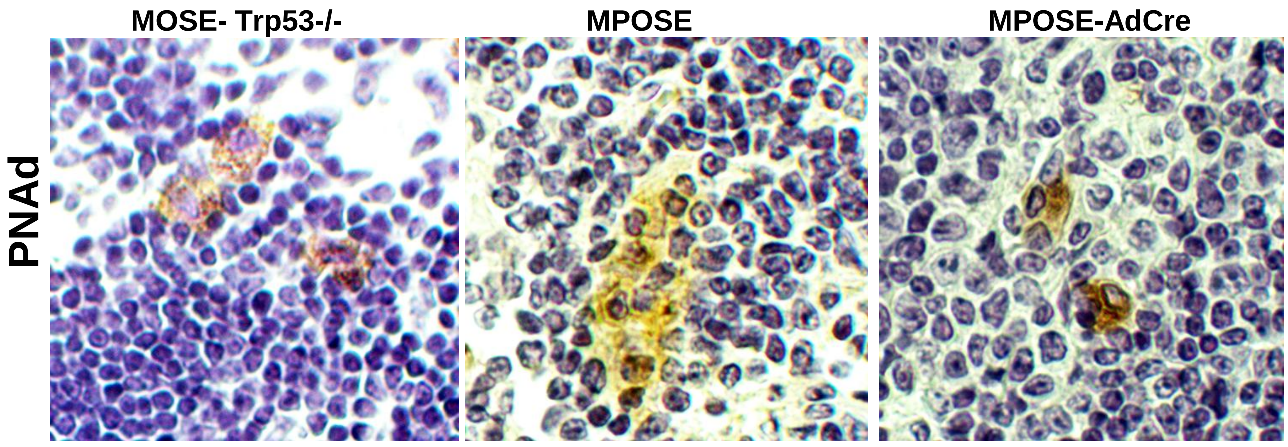

Fig S6. **Expression of peripheral node addressin (PNAd) in areas with intratumoral lymphocyte aggregates in three different tumor models.** IHC staining with mouse-specific PNAd antibody. Positive cells are shown in brown. Examples from one mouse, representative of at least 5 mice per model, are shown.

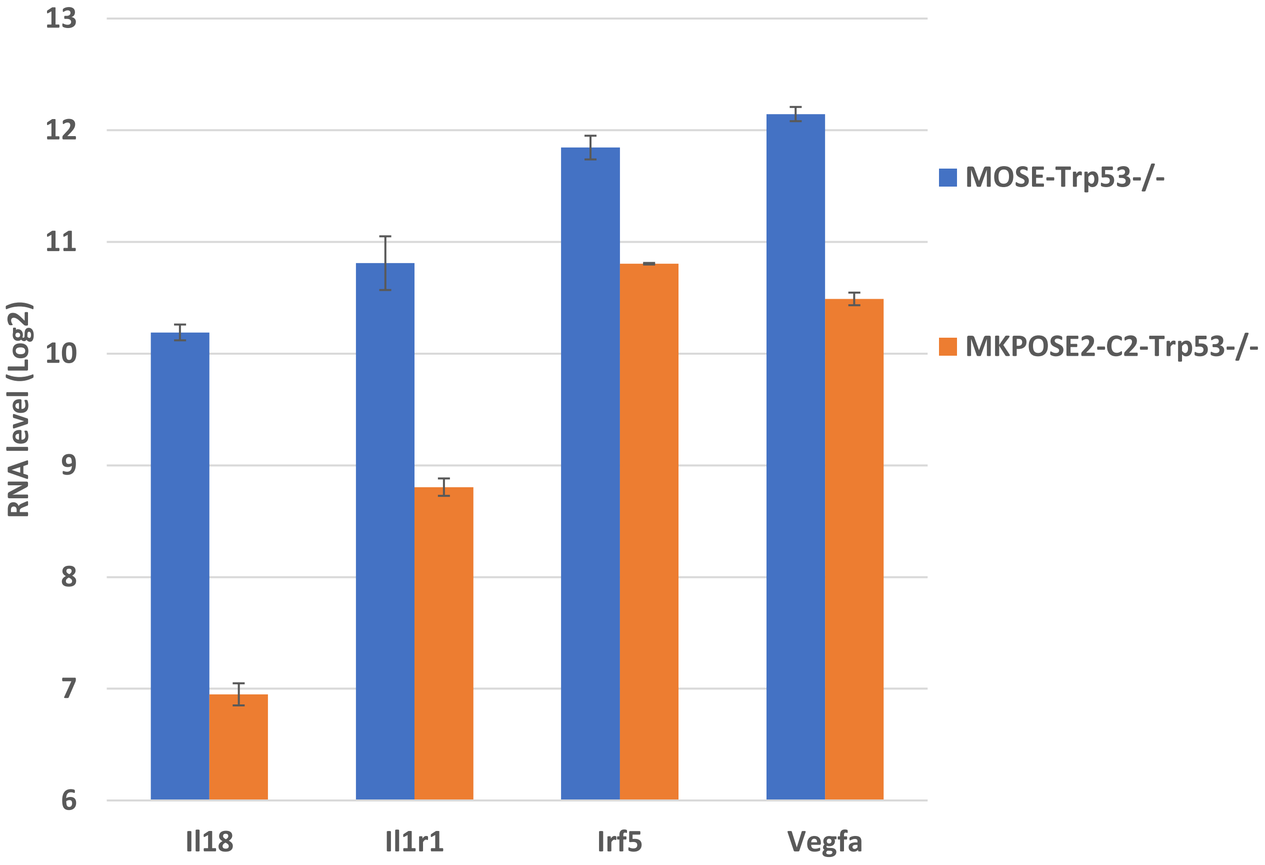

Fig S7. IL18, Il1r1, Lrf5, and Vegfa mRNA expression in MOSE-***Trp53***$^{-/-}$ cells and MKPOSE2-C2- ***Trp53***$^{-/-}$ cells.

Disease Models & Mechanisms • Supplementary information

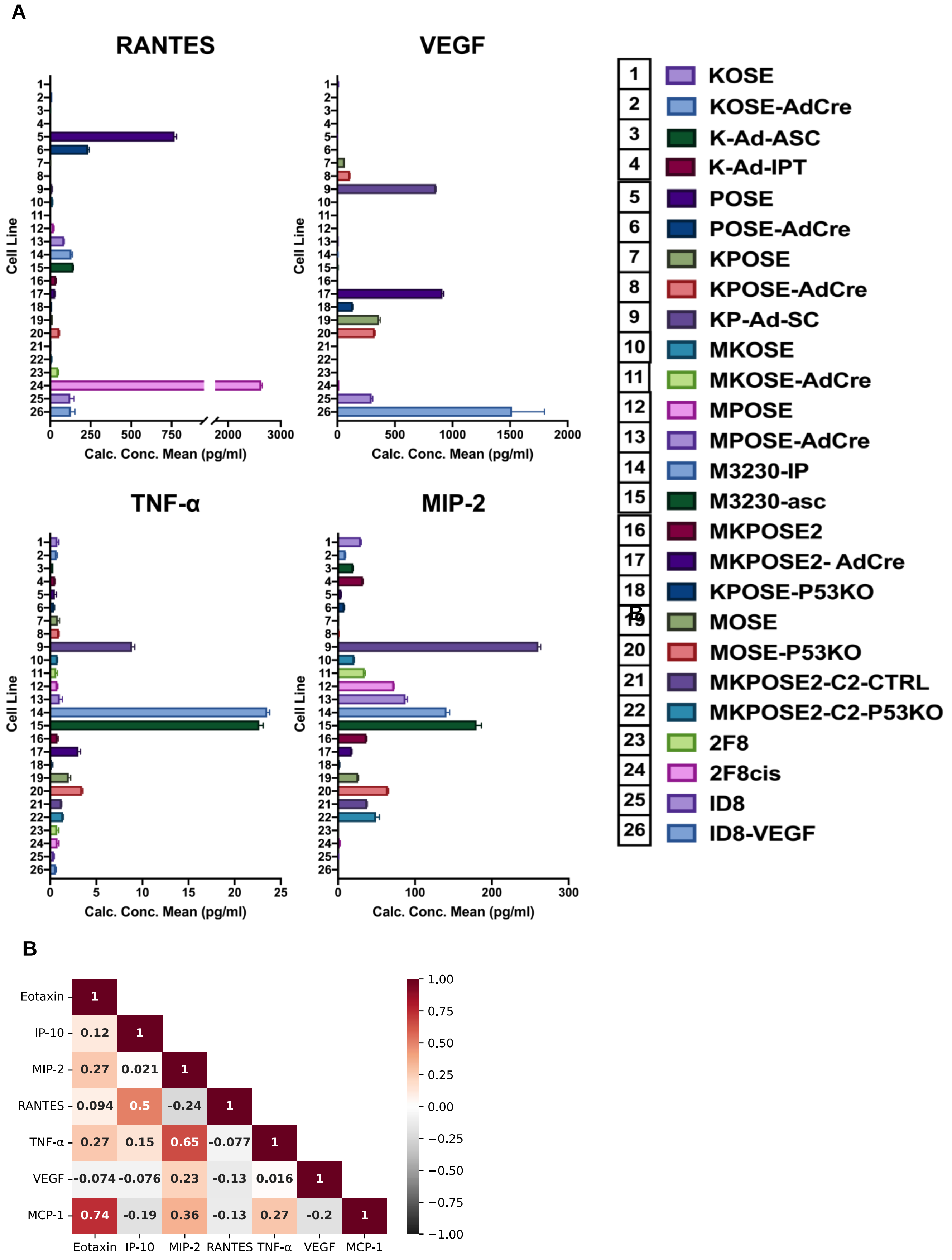

Fig S8. Cytokine and chemokine expression across 26 murine cell lines. (A) Cytokine and chemokines across 26 murine cell lines.(A) Murine RANTES, TNF α, MIP-2 and VEGF were measured in cell culture supernatants by MSD.. Values represent average concentration (in pg/ml) of two technical replicates. (B) Correlation heatmap. Scoring of +0.4 to +-0.6- moderate positive association; +0.6 to 0.8- strong positive association

Disease Models & Mechanisms • Supplementary information

Table S1. MOSE vs MOSE Trp53 null (n=367 DE genes, heatmap in Fig 4A)

Available for download at
https://journals.biologists.com/dmm/article-lookup/doi/10.1242/dmm.052177#supplementary-data

Table S2. MOSETrp53nulll vs MOSETrp53null IP Tumor (n=196 DE genes, heatmap in Fig. 4E)

Available for download at
https://journals.biologists.com/dmm/article-lookup/doi/10.1242/dmm.052177#supplementary-data

Table S3. Endometrioid versus HGSOC (n=360 DE genes, heatmap shown in Fig. 5J)

Available for download at
https://journals.biologists.com/dmm/article-lookup/doi/10.1242/dmm.052177#supplementary-data

Disease Models & Mechanisms • Supplementary information
